# Dual Excitation and Dual Emission in a 1,4‐Diazepine Bearing an Extended π‐System

**DOI:** 10.1002/chem.202501434

**Published:** 2025-06-25

**Authors:** Lars Killian, Ayla J. H. Dekker, Björn Grabbet, Sander J. W. Vonk, Martin Lutz, Freddy T. Rabouw, Arnaud Thevenon

**Affiliations:** ^1^ Organic Chemistry and Catalysis Institute for Sustainable and Circular Chemistry Faculty of Science Utrecht University Universiteitsweg 99 Utrecht 3584 CG The Netherlands; ^2^ Soft Condensed Matter & Biophysics Debye institute for Nanomaterials Science Faculty of Science Utrecht University Princetonplein 1 Utrecht 3584 CC The Netherlands; ^3^ Inorganic Chemistry and Catalysis Institute for Sustainable and Circular Chemistry Faculty of Science Utrecht University Universiteitsweg 99 Utrecht 3584 CG The Netherlands; ^4^ Structural Biochemistry Bijvoet Centre for Biomolecular Research Faculty of Science Utrecht University Universiteitsweg 99 Utrecht 3584 CG The Netherlands

**Keywords:** cyclodehydrogenation, dual emission, extended π‐system

## Abstract

We present the synthesis of a benzo[b]benzo[11,12]tetracene[5,6‐ef][1,4]diazepine (BBTDZ) and a benzo[g]phenanthro[9,10,1‐cde]indazolium (BPIZ), two novel N‐heterocyclic nanographenes. Both molecules were independently isolated from 2,3‐Dichloro‐5,6‐dicyano‐1,4‐benzoquinone (DDQ)‐mediated cyclodehydrogenation reaction of the previously reported benzo[f,g]tetracene β‐diketiminate (BT‐BDI). Our mechanistic investigation shows the instrumental role of Sc(OTf)_3_ in allowing for C─N bond formation over N─N bond formation. Unexpectedly, the BBTDZ compound shows unique dual emission and dual excitation (DE/DE) properties. A combination of spectroscopic and computational studies provides a rationalization of these features. Charge transfer followed by geometric relaxation were found to cause the dual, strongly red‐shifted emission. The understanding of both the reaction mechanism and mechanism of DE/DE can contribute to the rational design of materials with unique photophysical properties.

## Graphical abstract

Cyclodehydrogenation of a previously reported nanographene BDI leads to the formation of a 1,4‐diazepine via scandium‐catalyzed C‐H bond formation. This compound exhibits a unique combination of dual excitation and dual emission. The dual emission was found to be caused by a combination of charge transfer and geometric relaxation in the excited state.

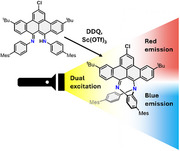

## 1 Introduction

Organic optoelectronic materials have received considerable attention over the past decades, due to their application in devices such as organic light‐emitting diodes (OLEDs) and sensors as well as their use in bioimaging.^[^ ^1^, ^2^ ^]^ Of particular interest are organic dual emitters—molecules in which two distinct emissive pathways are present simultaneously.^[^ ^3^ ^]^ Simultaneous emission from two excited singlet states violates Kasha's rule and is a relatively rare phenomenon in organic molecules. A number of different mechanisms are known by which dual emission manifests in organic molecules, including excited state intramolecular proton transfer (ESIPT),^[^ ^4^, ^5^ ^]^ excimer emission,^[^ ^6^, ^7^, ^8^ ^]^ and intramolecular charge transfer (ICT) processes.^[^ ^9^ ^]^ Despite their uncommon occurrence, these dual emitters are of high interest in materials chemistry, for example, for the fabrication of white organic light‐emitting diodes (WOLEDs).^[^ ^10^ ^]^ Apart from dual emission, large Stokes shifts are another highly desirable property in organic dyes, as they facilitate their use as laser gain materials and enable clear spectral separation of excitation and emission in imaging applications.^[^ ^11^, ^12^ ^]^ Molecular engineering of dyes which exhibit such large Stokes shifts has been accomplished by employing a range of strategies such as geometric relaxation in the excited state^[^ ^13^, ^14^ ^]^ and ICT processes, similar to the mechanisms by which dual emission is realized.^[^ ^11^, ^15^ ^]^ One class of molecules that has attracted much attention for its unique optical and electronic properties^[^ ^16^, ^17^, ^18^ ^]^ is that of polycyclic aromatic hydrocarbons (PAHs)—or nanographenes—which are defined by their extended π‐conjugated system.^[^ ^19^, ^20^, ^21^ ^]^ As a consequence of this extended π‐conjugation, they typically display narrow bandgaps and are highly redox active, being able to store multiple electron equivalents. The synthesis of nanographenes is often accomplished by oxidative cyclodehydrogenation reactions such as the Scholl reaction.^[^ ^22^ ^]^ Despite being known for over a century, much debate remains surrounding the mechanism of Scholl oxidation, with poor predictability as a result, hampering the rational design of PAHs with desired photophysical properties.^[^ ^23^ ^]^ Additionally, side reactivities such as halogenation^[^ ^24^, ^25^, ^26^ ^]^ and rearrangements^[^ ^27^ ^]^ are not uncommon, and the presence of heteroatoms^[^ ^21^, ^28^, ^29^ ^]^ can further complicate oxidative cyclodehydrogenation pathways, leading to unexpected reactivity.

**Scheme 1:**
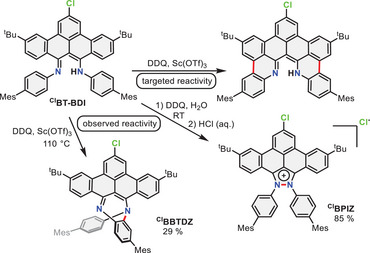
Targeted versus observed (divergent) oxidation reactivity of the **^Cl^BT‐BDI** ligand. New bonds are shown in red.

In recent work, we have reported on the synthesis of a novel π‐extended β‐diketiminate ligand, bearing a benzo[*f,g*]tetracene backbone (**BT‐BDI**). The synthesis was accomplished via a templated Scholl oxidation approach, in which two new carbon‐carbon bonds are formed to construct the benzo[*f*,g]tetracene framework. With this compound in hand, we aimed at forming the “remaining” two carbon‐carbon bonds (Scheme 1, top) using different Scholl oxidation conditions, since they did not form under the conditions used in the synthesis of **BT‐BDI** (FeCl_3_/MeNO_2_). However, the expected selectivity was not observed, and instead two different compounds could be isolated by changing the reaction temperature. Herein, we present the synthesis of these compounds, and our investigations into the mechanism of their formation as well as the dual emission and dual excitation (DE/DE) behavior of one of these compounds.

## 2 Results and Discussion

### 2.1 Synthesis and Mechanistic Investigations

We began our investigations into the further π‐extension of the previously reported compound **^Cl^BT‐BDI** ^[^ ^30^ ^]^ with the reaction of **^Cl^BT‐BDI** with one‐electron oxidant 2,3‐Dichloro‐5,6‐dicyano‐1,4‐benzoquinone (DDQ), and Sc(OTf)_3_ or methanesulfonic acid. Full conversion was observed at room temperature within 20 minutes, and the main product was isolated as a red‐purple solid using column chromatography. While in the ^1^H nuclear magnetic resonance (NMR) spectrum of the isolated material signals are broad, treating this compound with 4 M HCl (aq.) improves the sharpness of the signals so that the symmetry of the molecule can be clearly distinguished to be C_2v_, similarly to the starting material, **^Cl^BT‐BDI**. Further sharpening of the signals is accomplished by adding a drop of methanesulfonic acid and D_2_O to the NMR sample (in CDCl_3_), allowing for assignment of the signals in both ^1^H and ^13^C NMR spectra. Interestingly, this showed the same number of aromatic protons as in the starting material, implying no dehydrogenative C‐C bond formation took place. Sharpening of the broad NMR signals with the addition of acid suggests the presence of a cationic species, whose NMR signals are broadened by the presence of multiple different counter anions in the crude mixture. High‐resolution mass spectrometry (HRMS) measurements revealed the loss of 2 *m*/*z* compared to the starting material. Combined, these results point toward the formation of a pyrazolium‐type ring with a newly formed N‐N bond (Scheme 1, **^Cl^BPIZ**). Further optimization of the reaction conditions showed that the reaction does not need the (Lewis) acid additive, but water greatly accelerates the reaction, and it is best performed in a biphasic system (1/1 H_2_O/DCM), giving the isolated chloride salt, **[^Cl^BPIZ]Cl**, in an 85% yield. Similar oxidative N‐N bond formations are known, for example in the synthesis of 1,2,4‐triazoles^[^ ^31^, ^32^ ^]^ and the well‐known oxidative formation of tetrazoles from formazans, of which the reverse reaction is famously used in cell viability assays.^[^ ^33^ ^]^ An even more similar example of this type of oxidative N‐N bond formation was recently reported by Tonks et. al., who were able to transform diazatitanacycles—which show considerable similarity to titanium‐coordinated BDI ligands—into pyrazoles using 2,2,6,6‐tetramethylpiperidinyloxyl (TEMPO) as oxidant.^[^ ^34^, ^35^ ^]^

**Figure 1:**
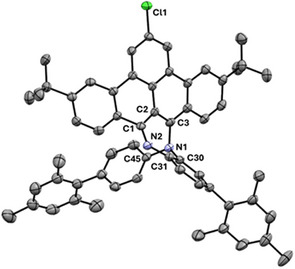
Displacement ellipsoid plot (50 % probability) of the asymmetric unit of **^Cl^BBTDZ**. Hydrogen atoms and severely disordered CH_2_Cl_2_ solvent molecules are omitted. Selected distances [Å] and angles [°]: C1‐C2 1.462(4), C2‐C3 1.367(4), C3‐N1 1.445(3), N1‐C30 1.439(3), C30‐C31 1.398(4), C31‐N2 1.401(3), N2‐C1 1.299(3), C3‐N1‐C30 109.3(3), C3‐N1‐C45 116.0(2), C30‐N1‐C45 116.3(2).

Interestingly, using the same reagents as those initially used for the synthesis of **^Cl^BPIZ**–namely DDQ and Sc(OTf)_3_ in toluene–another product can be formed as major product by refluxing the reaction mixture for 2 hours. The yellow solid that was isolated using column chromatography crystallized from a DCM/MeOH mixture. The crystal structure shows the formation of a C‐N bond between one of the 4‐mesityl aniline groups of the **^Cl^BT‐BDI** and the other BDI nitrogen, forming a 7‐membered 1,4‐diazepine ring (Scheme 1, Figure 1; **^Cl^BBTDZ**). The bond lengths in the 7‐membered ring reveal little delocalization with bond lengths alternating between single (e.g., C3‐N1, 1.445(3) Å) and double (e.g., C1‐N2, 1.299(3) Å) bonds. The benzo[*f,g*]tetracene backbone is twisted, with one nitrogen pointing up from the average benzo[*f,g*]tetracene plane, and one nitrogen pointing down from it. The crystal structure is in line with the solution state characterization by NMR spectroscopy, showing an asymmetric structure, as opposed to the C_2v_ symmetric starting material. In general, oxidative C‐N bond formation is a common strategy in the synthesis of N‐heterocycles.^[^ ^36^ ^]^ The formation of this particular C‐N bond is comparable to the C‐N bond formation under similar conditions we reported recently to form an indole‐type compound.^[^ ^30^ ^]^

The formation of such seeming kinetic (**^Cl^BPIZ**) and thermodynamic (**^Cl^BBTDZ**) products raises the question of whether direct conversion of **^Cl^BPIZ** to **^Cl^BBTDZ** is possible under certain reaction conditions. However, heating a sample of isolated **^Cl^BPIZ** in toluene with or without added Sc(OTf)_3_ and DDQ did not lead to any significant conversion. Furthermore, **^Cl^BPIZ** was not observed as an intermediate during the reaction to form **^Cl^BBTDZ** (Figure S22). This prompted us to further investigate this surprising divergent oxidative cyclodehydrogenation reactivity.

Conversion of **^Cl^BT‐BDI** was observed at room temperature using the standard reaction conditions (5 equiv. Sc(OTf)_3_ and DDQ in toluene). The observed compound is also observed without DDQ and has a distinct deep green color and a similar, albeit shifted pattern in the ^1^H NMR spectrum compared to the starting material (Figure S20). The moisture‐sensitive intermediate could be isolated in a N_2_‐filled glovebox, and single crystals were grown from a MTBE/hexane mixture at−40 °C. The crystal structure revealed a protonated ligand salt **[^Cl^BT‐BDI‐H]^+^** (Figure S35) with a [Sc(OTf)_4_(MeOH)_2_]^−^ counterion. It is likely that the protonation is a consequence of triflic acid impurities in the commercial Sc(OTf)_3_. The origin of MeOH in the structure is not entirely clear but might be a contamination from the **^Cl^BT‐BDI** starting material. The same color and similar ^1^H NMR spectra were also obtained when reacting **^Cl^BT‐BDI** with triflic acid or methanesulfonic acid, confirming that the compound formed in the reaction with Sc(OTf)_3_ is a protonated ligand salt (Figure S20). When water is added to the *in‐situ* prepared **[^Cl^BT‐BDI‐H]^+^** in the presence of DDQ, an instant color change from green to red is observed with the formation of **^Cl^BPIZ**. When mixing **^Cl^BT‐BDI** with DDQ in toluene alone is followed by ^1^H NMR spectroscopy, no notable conversion is observed. However, heating this sample at 100 °C leads to slow but direct conversion to **^Cl^BPIZ** over the course of multiple hours. This highlights the role of Sc(OTf)_3_, since heating a solution of **^Cl^BT‐BDI** in toluene with DDQ and with Sc(OTf)_3_ present as well leads to the formation of **^Cl^BBTDZ** over multiple hours as discussed for the general procedure for the synthesis of **^Cl^BBTDZ** (Figure S22). An overview of the investigated reaction conditions and the resulting product selectivities is shown in Scheme S1.

Since conversion to **^Cl^BPIZ** in the presence of DDQ is slow without the addition of water, we reason that water facilitates the oxidation of this compound by DDQ, possibly by stabilizing a (cationic) intermediate or by assisting with H‐atom transfer.

**Scheme 2:**
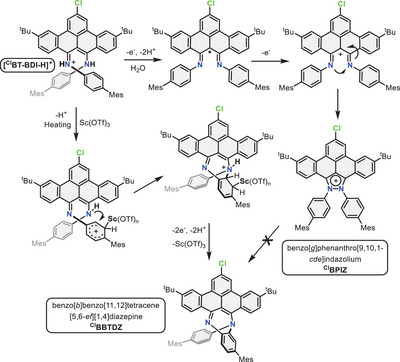
Proposed mechanism for the formation of ^**Cl**^ **BPIZ** and ^**Cl**^ **BBTDZ**. Reaction conditions: Sc(OTf)_3_ (5 equiv.), DDQ (5 equiv.), toluene.

Furthermore, reactions of **^Cl^BT‐BDI** with DDQ and triflic acid or methanesulfonic acid only led to the formation of **^Cl^BPIZ**, even after heating (Figure S21). Therefore, Sc(III) seems instrumental in allowing for the activation of the aniline carbon to form the C‐N bond in **^Cl^BBTDZ**. A plausible mechanism that takes all the above points into consideration is shown in Scheme 2. The coordination of Sc to the aryl ring is shown as η^1^. This hapticity is commonly observed for Sc(III)–aryl complexes^[^ ^37^, ^38^ ^]^ but the possibility of the intermediates proposed here adopting different binding modes/hapticities cannot be excluded.

### 2.2 Electrochemical and Photophysical Properties

The electrochemical properties of **^Cl^BPIZ**, **^Cl^BBTDZ** as well as **[^Cl^BT‐BDI‐H]^+^** were investigated using cyclic voltammetry (CV) and differential pulse voltammetry (DPV). CV of **[^Cl^BT‐BDI‐H]^+^** revealed that protonation shifts the peak potential for the first (irreversible) oxidation by 0.66 V in the anodic direction (Figure S30) compared to **^Cl^BT‐BDI**, showing that protonation initially hampers direct oxidation. This is in line with the mechanism discussed above, where heating (to form **^Cl^BBTDZ**) or water (to form **^Cl^BPIZ**) is needed to oxidize **[^Cl^BT‐BDI‐H]^+^**. CV of **^Cl^BPIZ** reveals little reversibility, with multiple irreversible oxidation and reduction events (Figure S27). Comparing this to the CV of **^Cl^BT‐BDI** ^[^ ^30^ ^]^ suggests that similar redox switching as known for tetrazoles and formazans^[^ ^33^ ^]^ is possible between **^Cl^BPIZ** and **^Cl^BT‐BDI** (Figure S29). In contrast, **^Cl^BBTDZ** shows one reversible reduction (−1.92 V versus Fc/Fc^+^) and one reversible oxidation (0.73 versus Fc/Fc^+^), as well as one quasi‐reversible oxidation (1.09 V versus Fc/Fc^+^). From the difference between the E_1/2_ of both reversible events, an electrochemical HOMO–LUMO gap of 2.65 eV was obtained (Figure S23–S26).

**Figure 2:**
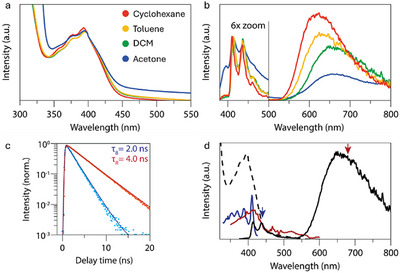
a) Absorption spectra of ^**Cl**^ **BBTDZ** in various solvents (25 µM). b) Emission spectra of ^**Cl**^ **BBTDZ** in various solvents (50 µM), normalized on the shortest wavelength emission peak (413 nm). Excitation at 350 nm. c) Photoluminescence decay curves for the red emission (680 nm) and blue emission (440 nm) of ^**Cl**^ **BBTDZ** in DCM (50 µM). d) Absorption (black dashed line), emission and excitation spectra of ^**Cl**^ **BBTDZ** in DCM. The color‐coded arrows indicate the wavelength at which the excitation spectra are recorded, shown in the corresponding color.

To investigate the photophysical properties of both new compounds, absorption and emission spectra were measured. **^Cl^BPIZ** shows maxima at 393 nm and 427 nm in dichloromethane (DCM), respectively (Figure S31). The absorption spectrum of **^Cl^BBTDZ** in DCM is similar, with an absorption maximum at 395 nm (Figure 2a). The emission spectrum, however, surprisingly shows two distinct emissions (Figure 2b). The first emission at 413 nm has a small Stokes shift of 1100 cm^−1^, while the other emission at 650 nm has a large redshift of 9930 cm^−1^. The presence of such a dual emission with one strongly red‐shifted emission prompted us to further investigate the origin of these uncommon features.

To confirm that the dual emission is an intrinsic property of the **^Cl^BBTDZ** molecule, we recorded emission spectra under various conditions. The blue and red emission are observed for a wide range of concentrations (10–250 µM) of **^Cl^BBTDZ** (Figure S32), demonstrating that the dual emission is not due to aggregation. The dual emission is also robust up to addition of up to 2 equivalents (5 µm–100 µM) of methanesulfonic acid (Figure S33), demonstrating that it is not due to an acid–base equilibrium. These reference experiments, combined with the high spectroscopic purity, the use of recrystallized samples for all photophysical measurements and the consistency over multiple batches show that the reported properties are indeed intrinsic to the **^Cl^BBTDZ** molecule.

Lifetime measurements of the emission from both states showed lifetimes of 2.0 ns and 4.0 ns for the blue and red emission, respectively, implying that both originate from singlet excited states (Figure 2c).^[^ ^39^ ^]^ A series of measurements in solvents of different polarity showed no influence of solvent polarity on the absorption maximum (Figure 2a), or the peak position of the blue emission (Figure 2b). The red emission, however, shows a redshift with more polar solvents, from 620 nm in cyclohexane to 667 nm in acetone (a shift of 1136 cm^−1^). Finally, excitation spectra for the red and blue emission are markedly different (Figure 2d). The excitation maxima at 410 nm largely overlap. However, the shape of the excitation spectrum for the blue emission is dominated by a vibrational progression extending toward shorter wavelengths, whereas the one for the red emission is broad and unstructured and extends to wavelengths as long as 570 nm. Whereas two emission bands, each a distinct excitation spectrum have been observed before, this is typically indicative of two different chemical species. For example, in certain porphyrinoids, double intramolecular hydrogen transfer (DIHT) gives two different tautomers with their own emission and excitation spectra.^[^ ^40^ ^]^ However, to the best of our knowledge, the combination of DE/DE under one set of conditions has not been observed before in cases where the two different states are related purely through geometric relaxation and/or charge transfer. To gain more insight into the origin of these remarkable photophysical properties, computational methods were used.

### 2.3 Rationalization of DE/DE

The geometries and electronics of the ground state (S_0_) and excited states (S_1_–S_5_) were calculated using conventional and time‐dependent DFT methods (see Supporting Information for computational details and discussion). Using DFT, a HOMO–LUMO gap of 3.24 eV (382 nm) was calculated, which is larger than obtained from electrochemistry (2.65 eV, vide supra), and than the estimate of the HOMO‐LUMO gap from the energy of the zero‐phonon line in the blue (410 nm, 3.02 eV). Optimizing the geometry of the first excited state (S_1_) shows significant relaxation compared to the ground‐state configuration. Upon relaxation, the HOMO–LUMO gap decreases to 2.36 eV (526 nm). The significant decrease of the HOMO–LUMO gap upon relaxation is consistent with the strongly redshifted emission band. In absolute numbers, the relaxed HOMO–LUMO gap in the experiment is again smaller (650 nm, 1.9 eV; from the peak energy of the red emission) than the calculated gap.

**Figure 3:**
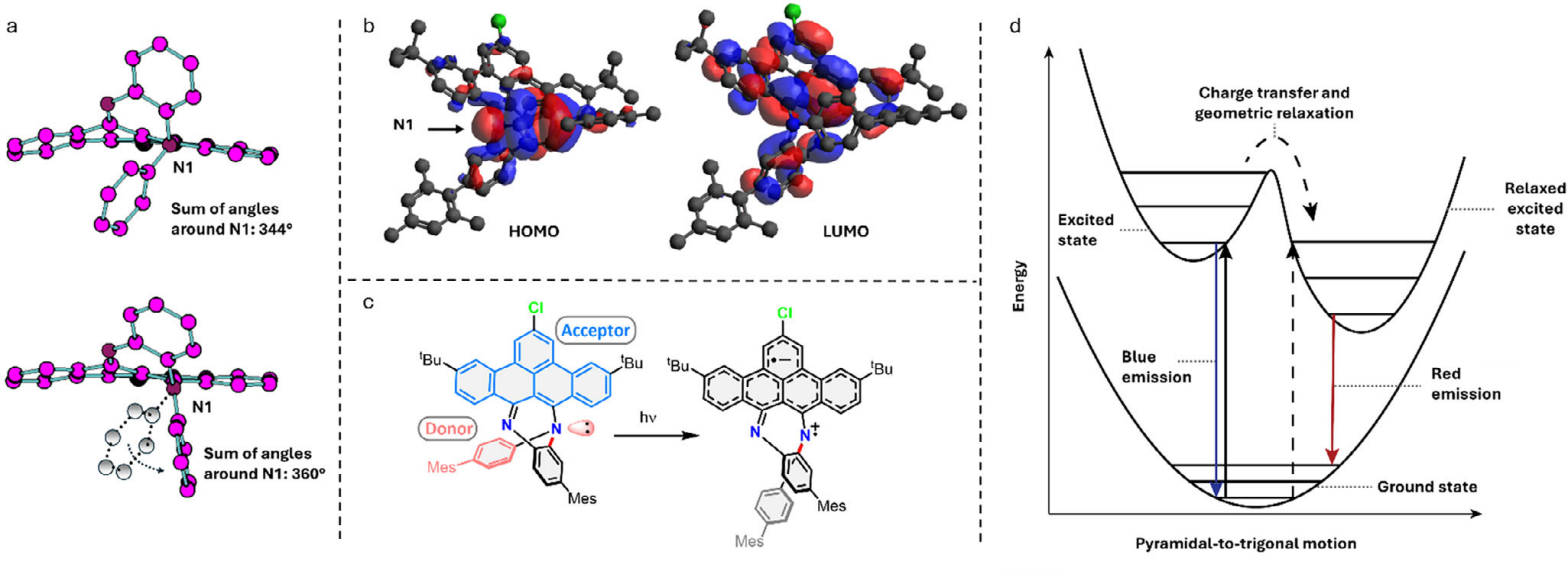
a) Optimized geometries of ^**Cl**^ **BBTDZ** in the ground state (top) and first excited state S_1_ (bottom), showing the geometric conversion of N1 from pyramidal to trigonal. Hydrogen atoms, mesityl groups, and tert‐butyl groups are not shown for clarity. b) Visualization of the HOMO (left) and LUMO (right) of ^**Cl**^ **BBTDZ**. c) Scheme showing the excited state charge transfer in ^**Cl**^ **BBTDZ**. d) Schematic of the excitations and emissions and their relative energies in relation to the motion around N1 in ^**Cl**^ **BBTDZ**.

To understand the origin of this smaller HOMO–LUMO gap in the relaxed S_1_ state we looked at the optimized geometries in more detail. The most striking difference between the relaxed S_0_ and S_1_ geometries is the geometry around the tertiary nitrogen N1. Whereas in the ground state, N1 is largely pyramidal with the sum of angles calculated at 344° (comparable to the 341.6(4)° in the crystal structure), in the relaxed S_1_ state the sum in angles is 360°, indicating the change to a trigonal geometry (Figure 3a). The change in geometry can be further explained by considering the nature of the excited state. Evaluating the frontier orbitals in the ground state, the HOMO consists largely of the lone pair on N1, conjugated to the aniline π‐system (Figure 3b). In the LUMO on the other hand, the electron density is delocalized over the benzo[*f*,g]tetracene π‐system. Therefore, HOMO–LUMO excitation constitutes charge transfer from the aniline donor unit to the benzo[*f*,g]tetracene acceptor (Figure 3c). This charge transfer from the nitrogen lone pair explains the change in geometry around N1, since a tertiary amine with a lone electron pair prefers a pyramidal local symmetry, while a tertiary amine with radical cation character prefers a trigonal symmetry. This charge transfer, combined with the geometric relaxation around N1 explains the strongly red‐shifted red emission. The solvent polarity dependence observed for the red emission can be understood as a stabilization of the charge‐separated excited state in more polar media. The observation of a combination of blue and red emission (Figure 3b) indicates an energy barrier for geometric relaxation. This barrier may kinetically hinder relaxation of the S_1_ excited state, leading to blue emission, or an excited state may cross the barrier, leading to red emission. This is illustrated in Figure 3d. If a two‐step process—optically excited charge transfer followed by geometric relaxation—were the only pathway toward red emission, identical excitation spectra would be expected for the blue and red emissions. Indeed, *N,N′‐*disubstituted‐dihydrodibenzo[*a,c*]phenazines–which are known to display similar dual emission through a combination of charge transfer and geometric relaxation–show identical excitation spectra of both emissions.^[^ ^14^ ^]^ In contrast, we observed different excitation spectra (*vide supra*). This indicates a direct excitation pathway from the ground state to the geometrically relaxed excited state. More precisely, it is consistent with the possibility of optical excitation in a geometry situated on the relaxed side of the energy barrier. Following this optical excitation, the molecule will undergo rapid further relaxation to the equilibrium geometry of the excited state before it emits red light. The straight, dashed arrow in Figure 3d shows this pathway for the excitation of red emission. The broadening of the red excitation and emission spectra corroborate the charge‐transfer character of this transition and the strong geometric relaxations associated.

## 3 Conclusion

In conclusion, we have reported on the synthesis of two new compounds via the divergent oxidative cyclodehydrogenation of a π‐extended β‐diketiminate (**^Cl^BT‐BDI**). Mechanistic investigations uncovered the origin of this divergent reactivity, highlighting the role of Sc(OTf)_3_ in allowing for C‐N bond formation over N‐N bond formation. These mechanistic insights contribute to the overall limited knowledge on the selectivity in oxidative cyclodehydrogenation and related reactions, especially concerning heteroatom involvement. One of the prepared compounds, ^**Cl**^ **BBTDZ** was shown to have unique photophysical properties, with strongly red‐shifted double emission as well as double excitation (DE/DE). These unique properties were investigated using a combination of spectroscopic and computational methods, giving a qualitative and semi‐quantitative explanation of the photophysical properties of ^**Cl**^ **BBTDZ**. More precisely, excited‐state relaxation through charge transfer and accompanying geometric relaxation was found to be the main reason for the strongly red‐shifted emission compared to the weakly Stokes‐shifted blue emission. The double excitation suggests that the relaxed excited state is additionally reached through direct charge transfer excitation from the electronic ground state. The understanding of these unique photophysical properties and the principles underlying them can help in the development of new materials and devices with applications in sensing, encryption^[^ ^41^ ^]^ bioimaging, and OLEDs, for example. We are currently investigating the use of more in‐depth spectroscopic and computational tools to provide a more comprehensive, quantitative model of the unique DE/DE properties of ^**Cl**^ **BBTDZ** first described herein.

## Supporting Information

Experimental procedures, characterization, spectroscopic data, electrochemical data, and computational details are available in the Supporting Information. Spectroscopic, electrochemical, and computational data files that support the findings of this study are openly available in the Yoda data repository at https://doi.org/10.24416/UU01‐5PNSDH. CCDC 2441497–2441498 contain the supplementary crystallographic data for this paper. These data can be obtained free of charge from The Cambridge Crystallographic Data Centre via www.ccdc.cam.ac.uk/data_request/cif. The authors have cited additional references within the Supporting Information.^[^ ^42^, ^43^, ^44^, ^45^, ^46^, ^47^, ^48^, ^49^, ^50^, ^51^, ^52^, ^53^, ^54^, ^55^, ^56^, ^57^, ^58^, ^59^, ^60^, ^61^, ^62^, ^63^, ^64^, ^65^, ^66^, ^67^, ^68^, ^69^, ^70^, ^71^ ^]^

## Conflict of Interest

The authors declare no conflict of interest.

## Supporting information



Supporting Information

## Data Availability

The data that support the findings of this study are openly available in Yoda repository at https://doi.org/10.24416/UU01‐5PNSDH, reference number 1744619180.
