# Supplementary material for: Dual Excitation and Dual Emission in a 1,4‐Diazepine Bearing an Extended π‐System
Source: Chemistry. 2025 Jun 25;31(50):e202501434. doi: 10.1002/chem.202501434 (PMC12415327; doi:10.1002/chem.202501434)
Supplement: Supplementary file 1 — Supporting Information [file CHEM-31-e202501434-s001.pdf]

Supplementary Information

Dual excitation and dual emission in a 1,4-diazepine bearing an extended π-system

Lars Killian[a], Ayla J.H. Dekker[+a,b], Björn Grabbet[+a], Sander J.W. Vonk[b,c], Martin Lutz[d], Freddy T. Rabouw[b,c], Arnaud Thevenon[*a]

[a] Organic Chemistry and Catalysis, Institute for Sustainable and Circular Chemistry, Faculty of Science, Utrecht University, Universiteitsweg 99, 3584 CG, Utrecht (The Netherlands)
[b] Soft Condensed Matter & Biophysics, Debye Institute for Nanomaterials Science, Faculty of Science, Utrecht University, Princetonplein 1, 3584CC, The Netherlands
[c] Inorganic Chemistry & Catalysis, Institute for Sustainable and Circular Chemistry, Faculty of Science, Utrecht University, Universiteitsweg 99, 3584 CG, The Netherlands
[d] Structural Biochemistry, Bijvoet Centre for Biomolecular Research, Faculty of Science, Utrecht University, Universiteitsweg 99, 3584 CG, Utrecht (The Netherlands)
[+]These authors contributed equally
*Corresponding author. E-mail: a.a.thevenon-kozub@uu.nl

S1 General remarks 3
S2 Synthesis and characterisation 5
S3 NMR spectra 8
S4 Electrochemistry 20
S5 UV-Vis and PL spectroscopy 24
S6 X-ray structure determination 26
S7 IR spectra 30
S8 HRMS spectra 31
S9 Computational results 32
References 43

S1 General remarks

Unless otherwise stated, commercial reagents and solvents were used as received. **^{Cl}BT-BDI** was synthesized according to a previously reported protocol.[1]

NMR measurements were performed at 25 °C on a Varian VNMRS400, or a Jeol JNMECZL G 400 MHz spectrometer. Chemical shifts in ^{1}H and ^{13}C are reported relative to TMS with the residual solvent signal[2] as internal standard where the influence of analytes on the solvent can be assumed negligible. Peak multiplicity is quoted as s (singlet), bs (broad singlet), d (doublet), t (triplet) and so on.

FT-IR data was recorded on a PerkinElmer SpectrumTwo Infrared Spectrophotometer with an ATR probe.

HRMS was measured on an Agilent Technologies 6560 ion mobility QTOF using direct infusion.

Electrochemical measurements were performed in a N2-filled MBraun labmaster dp glovebox, using an IVIUM potentiostat/galvanostat. A three-electrode set-up was used with Ag/$AgNO_3$ (saturated solution in electrolyte) reference electrode, Pt wire counter electrode and glassy carbon (3 mm Ø) working electrode directly in solution. All electrochemical data is referenced to the ferrocene/ferrocenium couple, measured on the same day as the experiment.

Column chromatographic purifications were done either using standard techniques and glassware or using the Büchi Pure C-810 Flash with UV-detector in combination with Büchi FlashPure EcoFlex silica columns.

Emission and excitation spectra were recorded on 50 μM solutions in quartz cuvettes using an Edinburgh Instruments Inc FLS920 spectrophotometer with TMS300 monochromators and a R928 photomultiplier tube. A 450 W xenon arc lamp was used as excitation source.

UV-Vis absorption spectra were obtained on 25 μM solution in quartz cuvettes using a LAMBDA 365 UV/Vis Spectrophotometer.

Lifetime decay measurements were performed on a home-built optical setup consisting of a Nikon Ti-U inverted microscope body on the molecule in a 50 μM solution in a glass vial. A 405-nm pulsed laser (Picoquant D-C 405, controlled by Picoquant PDL 800-D laser driver) was guided to the sample by a dichroic mirror (edge at 425 nm, Thorlabs DMLP425R) and focused by a 40× Nikon CFI Plan Fluor (NA = 0.75) air objective into the sample. The emission was collected by the same objective and guided to an avalanche photodiode (APD; Micro Photonic Devices PDM). To select the blue emission, a 450 nm short-pass (Thorlabs FESH450) filter was used. For the red emission, a 600 nm long-pass (Thorlabs FELH600) filter was used.

All calculations were carried out using Density Functional Theory (DFT)[3] and Time-dependent-DFT (TD-DFT)[4–6] as implemented in Gaussian Version 16.01.[7] Geometry optimizations and frequency analyses were performed with the B3LYP[8–11] functional including Grimme's D3 dispersion correction with Becke-Johnson Damping[12] with the extended 6-311+G(d,p) Pople-style basis set (synonymously to 6-311+G**)[13–19], together with an applied pseudosolvation by the in Gaussian integrated Polarizable Continuum Model (PCM).[20,21] Nature of calculated geometries were evaluated by frequency analysis, confirming no imaginary frequencies and low gradients. Results were visualized with either Chemcraft[22], *Avogadro 1.2.0*[23] or *MultiWFN 3.8.*[24]

Spectroscopic, electrochemical, and computational data files that support the findings of this study are openly available in the Yoda data repository at https://doi.org/10.24416/UU01-5PNSDH.

S2 Synthesis and characterisation

^{Cl}BBTDZ

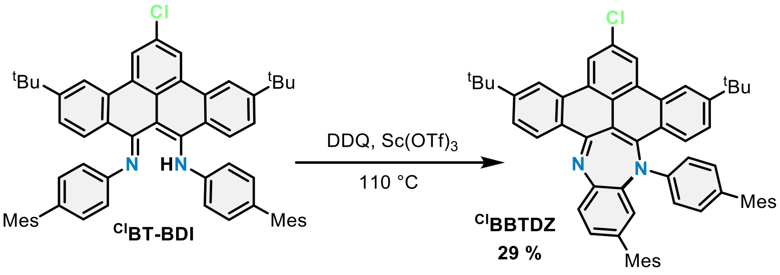

To a 100 mL round bottom flask were added a magnetic stir bar, **^{Cl}BT-BDI** (52.4 mg, 0.063 mmol, 1 equiv.), DDQ (71.7 mg, 0.32 mmol, 5 equiv.) and $Sc(OTf)_3$ (135.5 mg, 0.32 mmol, 5 equiv.) followed by dry toluene (15 mL). Within several minutes, the mixture turned from reddish-brown to dark green. After 30 minutes stirring at room temperature, the mixture was refluxed for 2h, in which the colour changed to reddish-brown. After cooling down to room temperature, water (40 mL) was added, and the organics were extracted with toluene (40 mL). The organics were washed with water (2 x 40 mL) and brine (40 mL), filtered over celite and the volatiles were removed under reduced pressure to leave a brown solid. **^{Cl}BBTDZ** was isolated using column chromatography on silica using a gradient eluent (PE / EtOAc 0→100 %). The isolated yellow fraction was recrystallized by slow evaporation of MTBE from a MTBE/MeOH mixture giving **^{Cl}BBTDZ** as yellow to orange crystals (15.2 mg, 29 %).

^{1}H NMR (400 MHz, $CDCl_3$, 298 K): δ = 8.75 (d, $^4J_{H,H}$ = 2.1 Hz, 1H), 8.69 (d, $^4J_{H,H}$ 1.4 Hz, 1H), 8.61 (s, 1H), 8.59 (s, 1H), 7.80 ($^3J_{H,H}$ = 8.6 Hz, $^4J_{H,H}$ = 1.8 Hz, 1H), 7.77 (d, $^3J_{H,H}$ = 8.2 Hz, 1H), 7.60 (dd, ($^3J_{H,H}$ = 8.3 Hz, $^4J_{H,H}$ = 1.8 Hz, 1H), 7.53 ($^4J_{H,H}$ = 1.9 Hz, 1H), 7.23 (dd, $^3J_{H,H}$ = 8.1, $^4J_{H,H}$ = 1.8 Hz, 1H), 7.02 (s, 1H), 6.93 (s, 1H), 6.84 (s, 2H), 6.69 (d, $^3J_{H,H}$ = 8.8 Hz, 2H), 6.40 (d, $^3J_{H,H}$ = 8.7 Hz, 2H), 2.35 (s, 3H), 2.29 (s, 3H), 2.26 (s, 3H), 1.90 (s, 6H), 1.87 (s, 3H), 1.52 (s, 9H), 1.47 (s, 9H) ppm.

^{13}C{^{1}H} NMR (101 MHz, $CDCl_3$, 298 K): δ = 157.5, 153.7, 152.8, 146.7, 144.4, 142.5, 142.0, 139.1, 137.9, 137.3, 137.2, 136.7, 136.3, 136.2, 136.0, 133.6, 133.3, 132.6, 132.0, 131.7, 131.7, 131.2, 130.3, 130.1, 129.6, 129.3, 128.9, 128.5, 128.4, 128.0, 127.2, 127.0, 126.7, 125.5, 125.4, 123.2, 122.3, 122.0, 119.4, 119.3, 112.2, 35.7, 35.4, 31.6, 31.5, 21.3, 21.2, 21.1, 21.1, 20.9 ppm.

ATR-IR: ν = 2962 (s), 2912 (m), 2867 (m), 1609 (m), 1564 (m), 1500 (m), 1467 (m), 1402 (m), 1361 (m), 1292 (s), 1260 (s), 1079 (m), 1026 (m), 932 (w), 845 (m), 803 (m), 735 (m), 567 (w) cm^{-1}.

HR-MS: m/z = 827.4184 {$[M+H]^+$, calc. 827.4132}.

Note: A DCM/MeOH mixture works well as an alternative solvent mixture for recrystallisation.

^{Cl}BPIZ$^+$

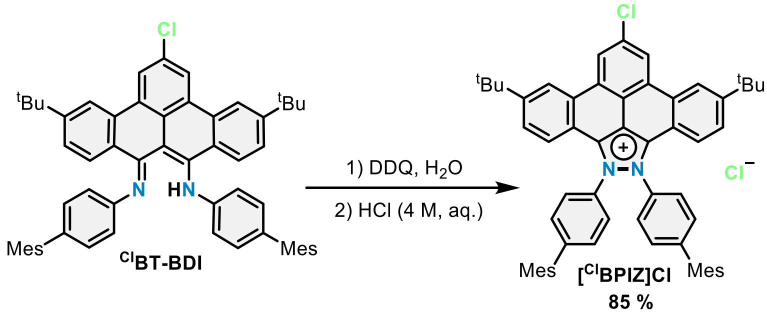

To a 100 mL round bottom flask were added a magnetic stir bar and 6 mL H_2O. **^{Cl}BT-BDI** (29.5 mg, 0.036 mmol, 1 equiv.) and DDQ (40.3 mg, 0.18 mmol, 5 equiv.) in DCM (6 mL) were added. After stirring for 40 minutes at room temperature, the organic and aqueous phases were separated, and the organics were extracted with DCM (5 mL). The organics were washed with water (3 x 5 mL) and the volatiles were removed under reduced pressure to leave a reddish solid. **^{Cl}BPIZ** was isolated using column chromatography on silica using a gradient eluent (PE / EtOAc, 0 → 100 %). The isolated purple fraction was dissolved in DCM (5 mL) and HCl (4 M aqueous, 5 mL) was added. The mixture was stirred overnight, after which the layers were separated, the organics were extracted with DCM (5 mL) and washed with water (3 x 5 mL). After removing the volatiles under reduced pressure, **[^{Cl}BPIZ]Cl** was isolated as a purple solid (26 mg, 85 % based on full conversion to the chloride salt).

^{1}H NMR (400 MHz, $CDCl_3$, 298 K, added drop of D_2O and $MeSO_3H$): δ = 8.87 (s, 2H), 8.85 (d, $^4J_{H,H}$ = 1.9 Hz, 2H), 8.07 (d, $^3J_{H,H}$ = 8.4 Hz, 4H), 7.63 (dd, $^3J_{H,H}$ = 8.8, $^4J_{H,H}$ = 1.8 Hz, 2H), 7.47 (d, $^3J_{H,H}$ = 8.7 Hz, 2H), 7.46 (d, $^3J_{H,H}$ = 8.4 Hz, 4H), 7.02 (s, 2H), 6.96 (s, 2H), 2.36 (s, 6H), 2.17 (s, 6H), 1.84 (s, 6H), 1.54 (s, 18H) ppm.

^{13}C{^{1}H} NMR (101 MHz, $CDCl_3$, 298 K, added drop of D_2O and $MeSO_3H$): δ = 155.9, 146.7, 137.9, 137.6, 136.8, 135.3, 135.1, 134.9, 133.9, 132.0, 131.6, 131.1, 129.4, 128.7, 128.5, 127.5, 124.5, 124.0, 122.2, 117.4, 116.9, 36.0, 31.4, 21.2, 20.9, 20.6 ppm.

ATR-IR: ν = 2961 (s), 2921 (m), 2870 (m), 1589 (m), 1568 (s), 1512 (s), 1479 (m), 1448 (m), 1379 (m), 1263 (m), 1204 (w), 1099 (w), 1019 (w), 856 (w), 816 (w), 798 (w), 633 (w), 597 (w), 562 (w) cm^{-1}.

A small, sharp peak is observed at 2208 cm^{-1}. This corresponds to the nitrile group of DDQ, which is likely present as counterion (DDQ^-) even after treatment with aqueous HCl. In the ^{13}C NMR spectrum however, no peaks corresponding to such a counterion are observed, so we reason it is present only in small amounts.

HR-MS: m/z = 827.4132 {[M]$^+$, calc. 827.4132}.

An additional signal is observed at m/z = 849.5142. This corresponds to the calculated mass of the analogous *tert*-butyl substituted compound {[M]$^+$, calc. 849.5148}, derived from **^{tBu}BT-BDI**.[1] NMR spectroscopic analysis (Figure S7) shows a minor impurity with similar chemical shifts that might correspond to this compound.

[^{Cl}BT-BDI-H][Sc(OTf)$_4$(MeOH)$_2$]

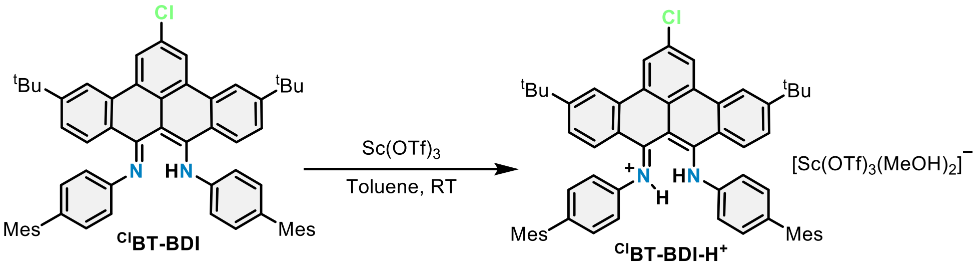

In an N_2-filled glovebox, **^{Cl}BT-BDI** (19.8 mg, 0.024 mmol, 1 equiv.) and Sc(OTf)$_3$ (51.2 mg, 0.12 mmol, 5 equiv.) were mixed in a vial with toluene (6 mL) and stirred at room temperature for 40 minutes. After mixing, the colour changes over the course of several minutes from red to green. Then, the volatiles were evaporated *in vacuo*, and the green solids were washed with hexane (3 mL) and extracted with DCM (5 mL). After removing the DCM *in vacuo*, **[^{Cl}BT-BDI-H][Sc(OTf)$_3$(MeOH)$_2$]** was obtained as a dark green solid (36.1 mg, quantitative yield (see note)).

Note: The determined yield given here amounts to 108 %, based on the counterion found in the crystal structure being the same as that of the bulk material. However, we do not exclude variations in the counterion in the bulk material compared to the crystal. This is further supported by the fact that methanol (present in the crystal structure) does not seem present in significant amounts in the ^{1}H or ^{13}C NMR spectrum. The compound can be further purified by crystallisation from MTBE/hexane at -40 °C.

Spectroscopic data is given to support the presence of a protonated **^{Cl}BT-BDI-H$^+$**, because of its importance in the mechanism of the reactions presented in the main text. Otherwise, limited spectroscopic data is provided.

The compound is somewhat stable towards most non-dried solvents and manipulations under ambient conditions, like those used in the synthesis of **^{Cl}BBTDZ** and **^{Cl}BPIZ**, in which **^{Cl}BT-BDI-H$^+$** is an intermediate. Over time however, decomposition under these conditions does take place.

^{1}H NMR (400 MHz, toluene-*d$_8$*, 298 K): δ = 10.39 (s, 2H), 8.68 (s, 2H), 8.36 (s, 2H), 8.02 (s, 2H), 7.59 (d, $^3J_{H,H}$ = 6.7 Hz, 2H), 7.23 (s, 4H), 6.91 (d, $^3J_{H,H}$ = 8.7 Hz, 2H), 2.21 (s, 6H), 2.03 (s, 12H), 1.23 (s, 18H) ppm.

^{13}C{^{1}H} NMR (101 MHz, toluene-*d$_8$*, 298 K): δ = 157.7, 141.5, 138.1, 136.9, 135.6, 135.2, 131.7, 131.5, 128.7, 124.5, 123.0, 120.4, 102.9, 35.6, 30.7, 21.1, 20.7 ppm.

^{19}F NMR (376 MHz, toluene-*d$_8$*, 298 K): δ = -77.1 (s) ppm.

S3 NMR spectra

^{Cl}BBTDZ

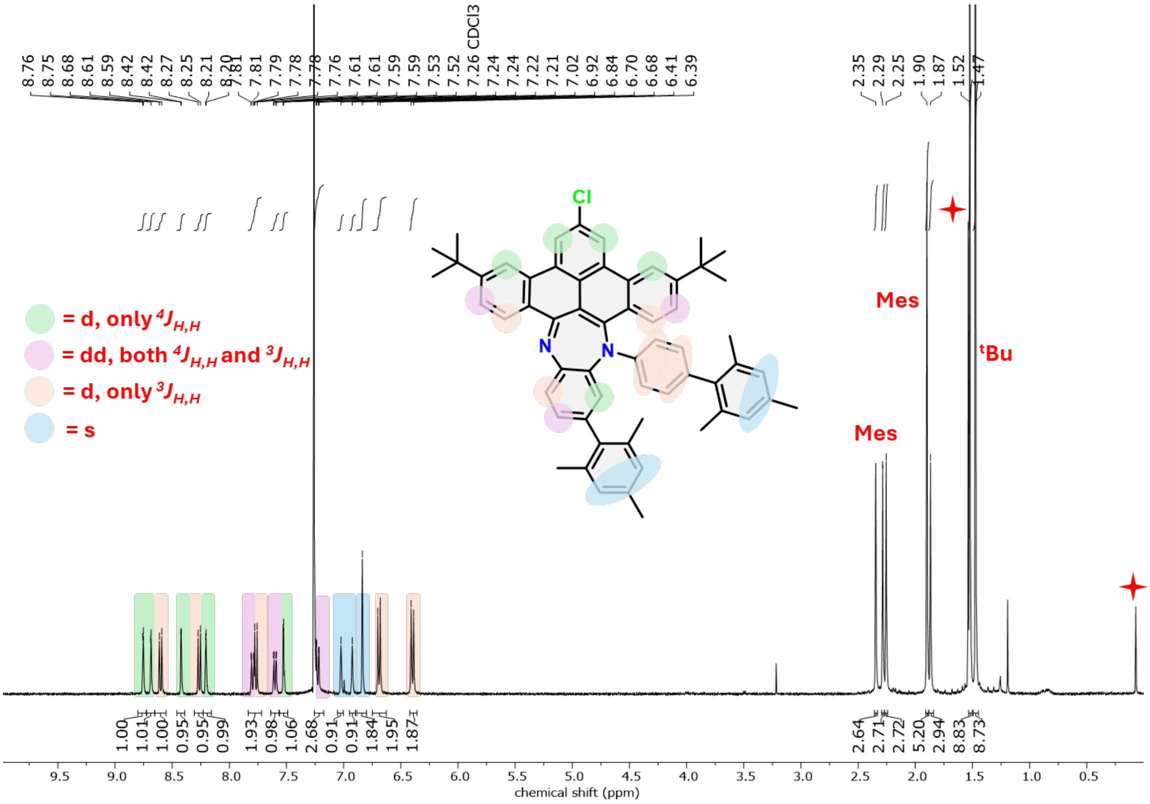

Figure S1: ^{1}H NMR spectrum of ^{Cl}**BBTDZ** in $CDCl_3$, at 25 °C. The resonances marked with a star are attributed to residual water in the solvent (1.56 ppm) and laboratory grease (0.08 ppm).

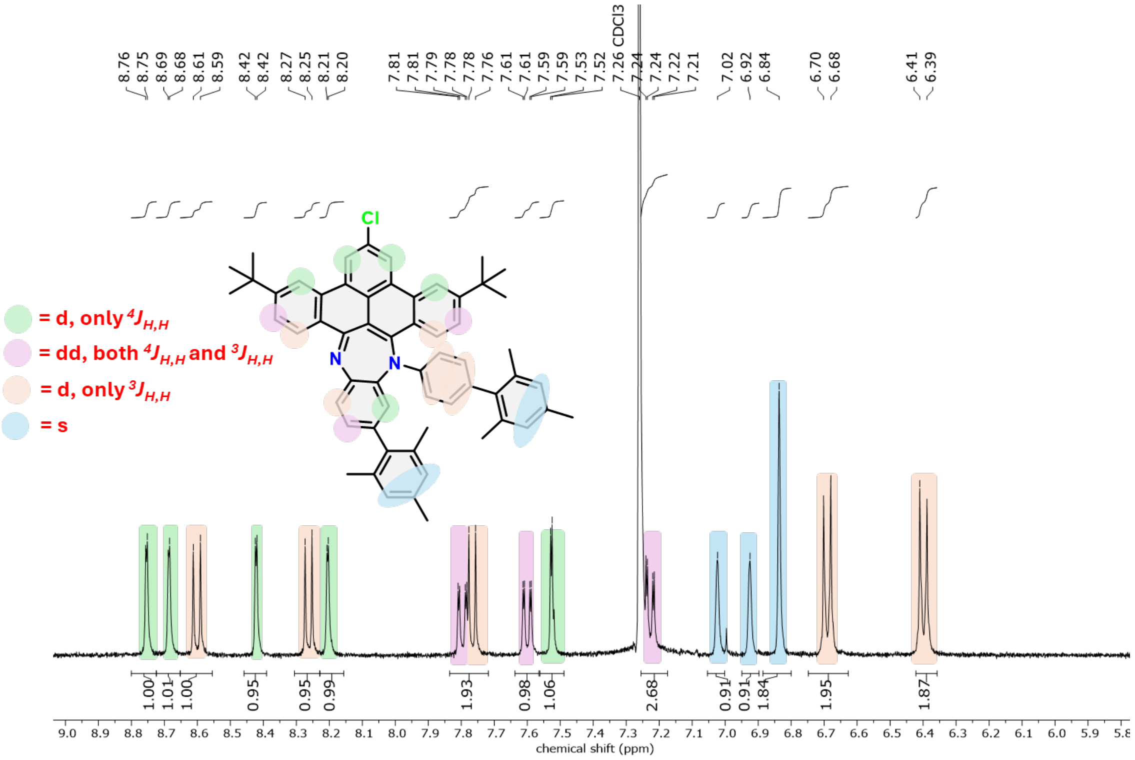

Figure S2: ^{1}H NMR spectrum (aromatic region) of ^{Cl}**BBTDZ** in $CDCl_3$, at 25 °C.

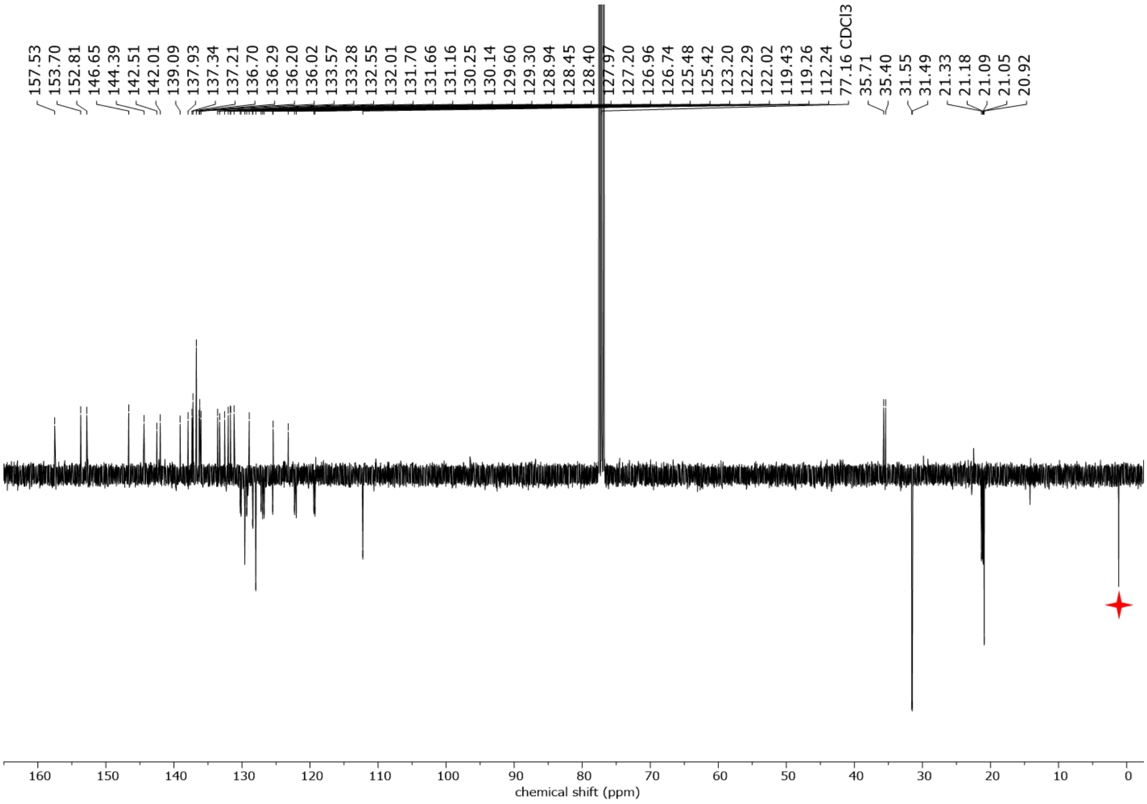

Figure S3: ^{13}C-APT NMR spectrum of ^{Ct}**BBTDZ** in $CDCl_3$, at 25 °C. The resonance marked with a star is attributed to residual laboratory grease.

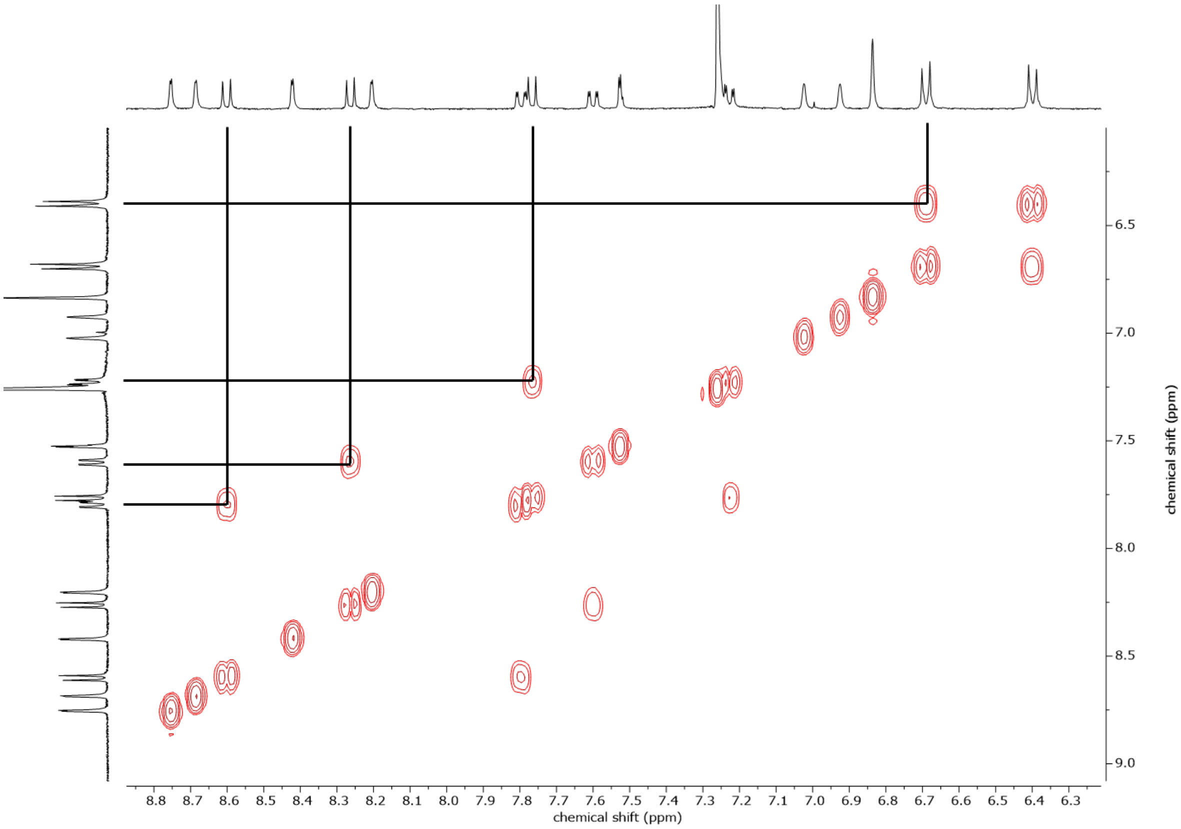

Figure S4: COSY NMR spectrum of ^{Ct}**BBTDZ** in $CDCl_3$, at 25 °C.

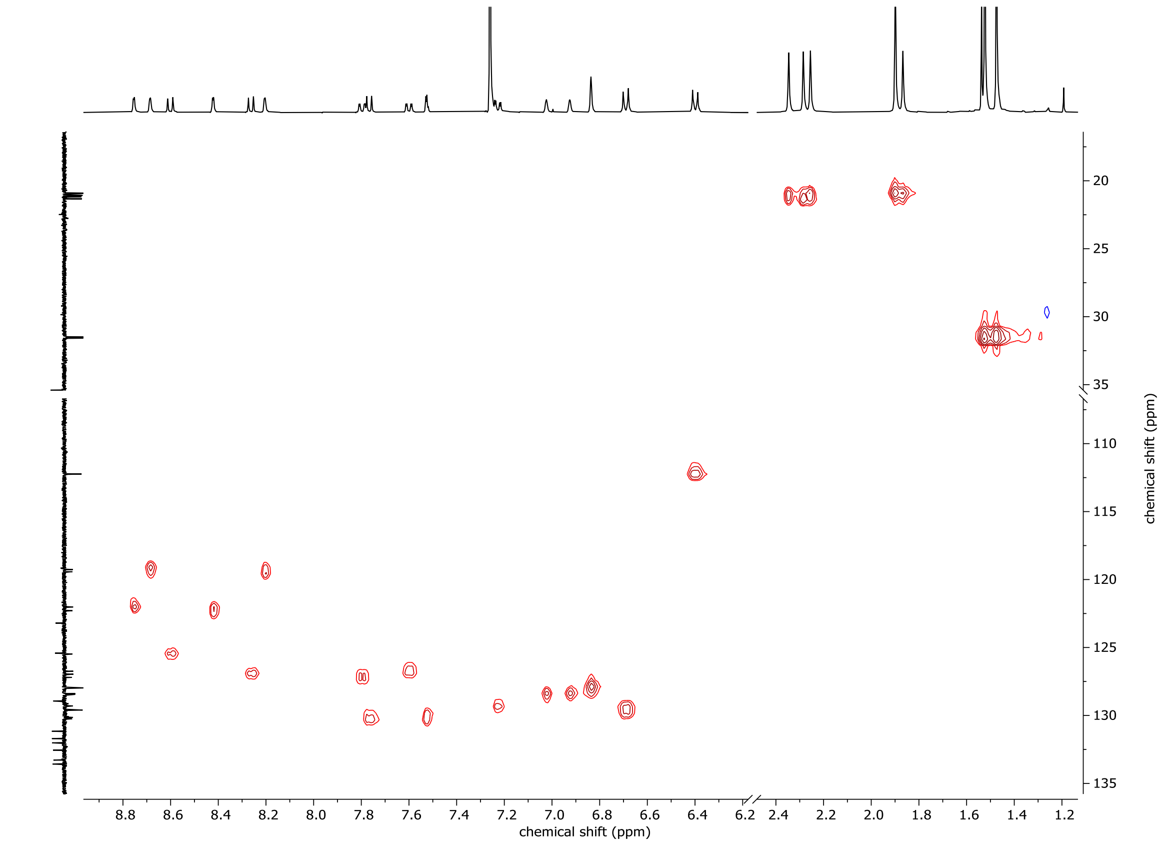

Figure S5: ^{1}H-^{13}C HSQC NMR spectrum of ^{Cl}**BBTDZ** in $CDCl_3$, at 25 °C.

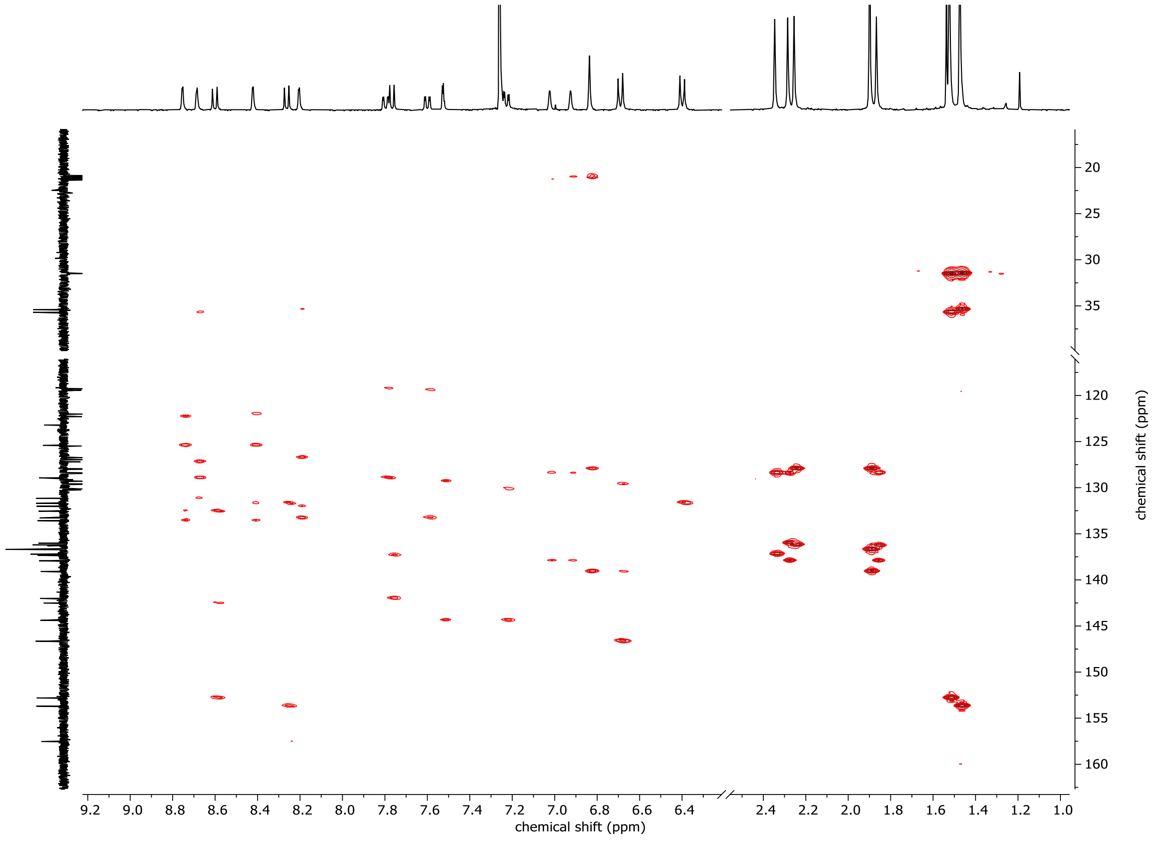

Figure S6: ^{1}H-^{13}C HMBC NMR spectrum of ^{Cl}**BBTDZ** in $CDCl_3$, at 25 °C.

^{Cl}**BPIZ**

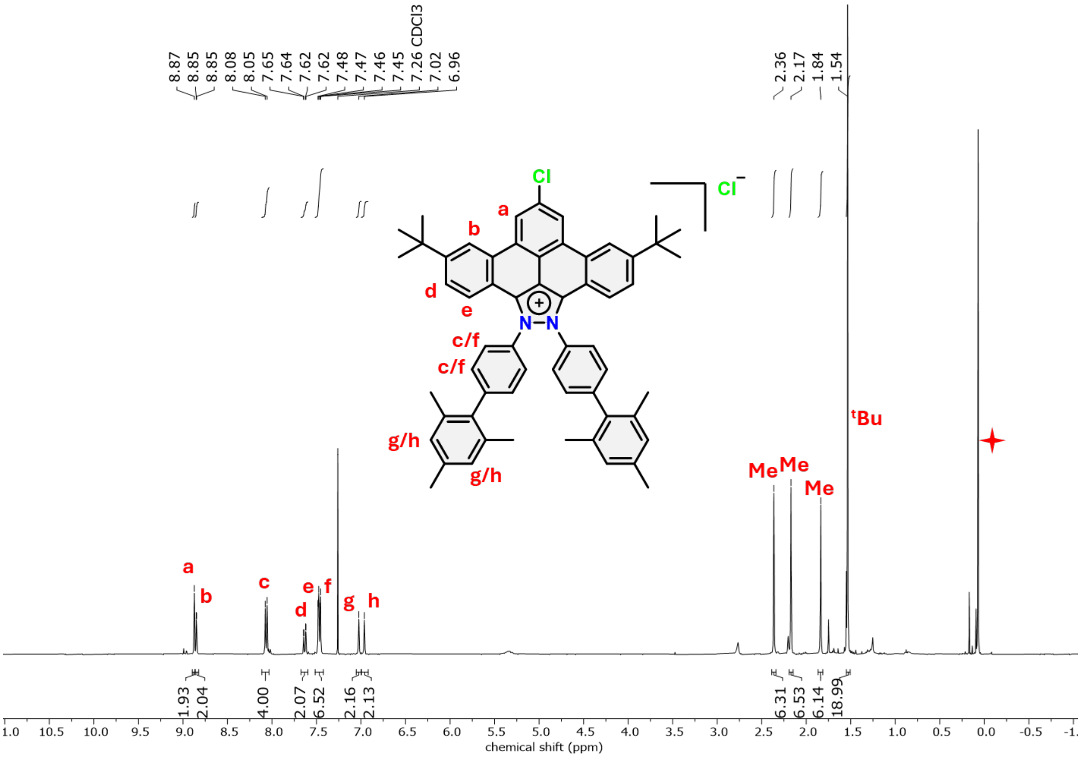

Figure S7: ^{1}H NMR spectrum of **[^{Cl}BPIZ]Cl** in $CDCl_3$, with a drop of D_2O and $MeSO_3H$ at 25 °C. The resonance marked with a star is attributed to residual laboratory grease.

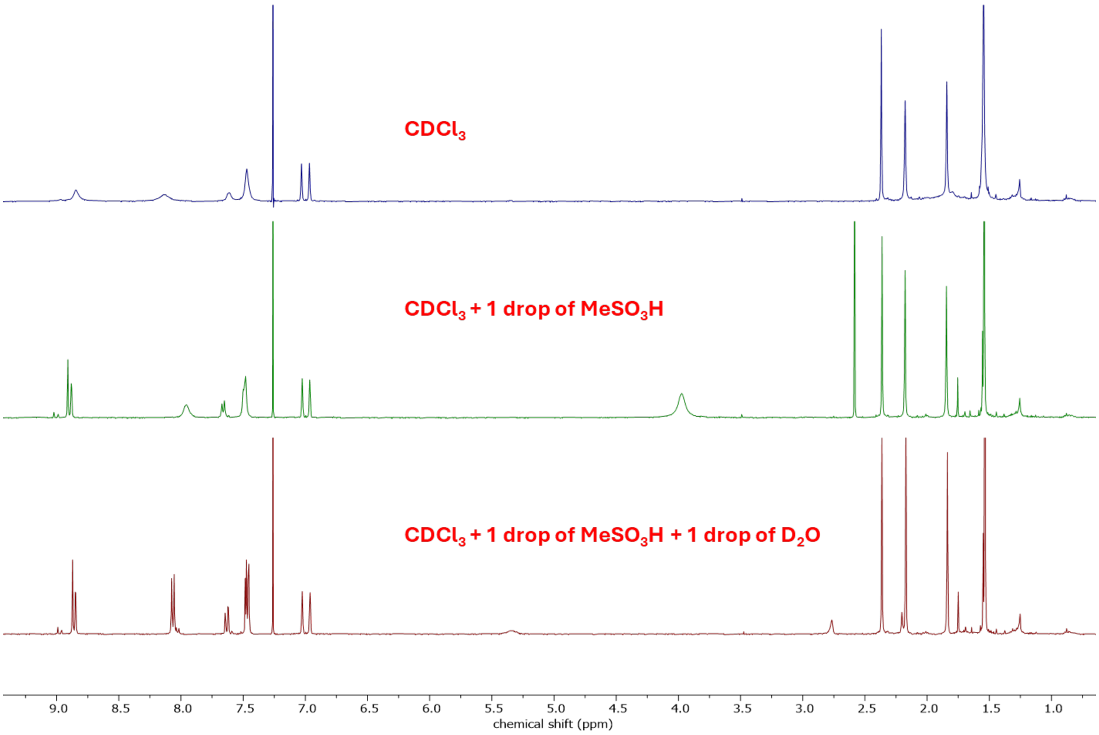

Figure S8: Stacked ^{1}H NMR spectra of **[^{Cl}BPIZ]Cl** in $CDCl_3$ at 25 °C, showing the sharpening of the signals upon addition of $MeSO_3H$ and D_2O.

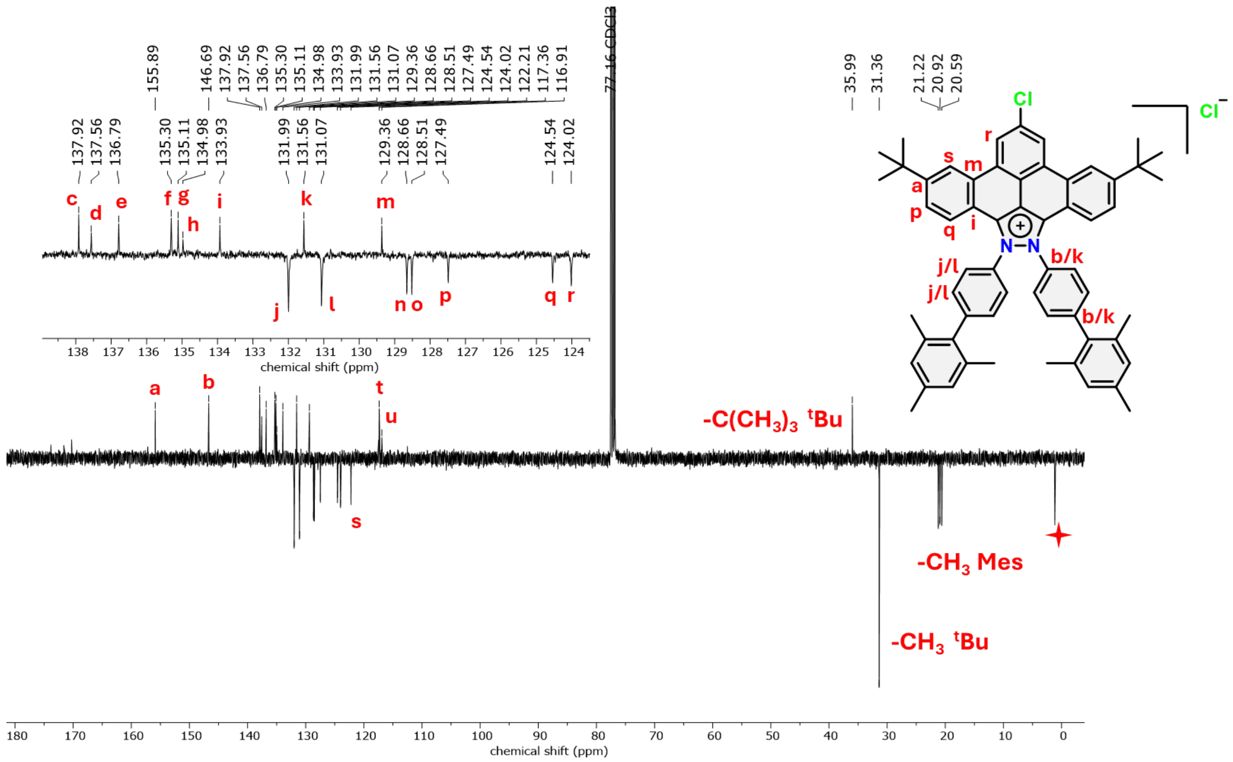

Figure S9: ^{13}C NMR spectrum of **[^{Cl}BPIZ]Cl** in $CDCl_3$, with a drop of D_2O and $MeSO_3H$ at 25 °C.

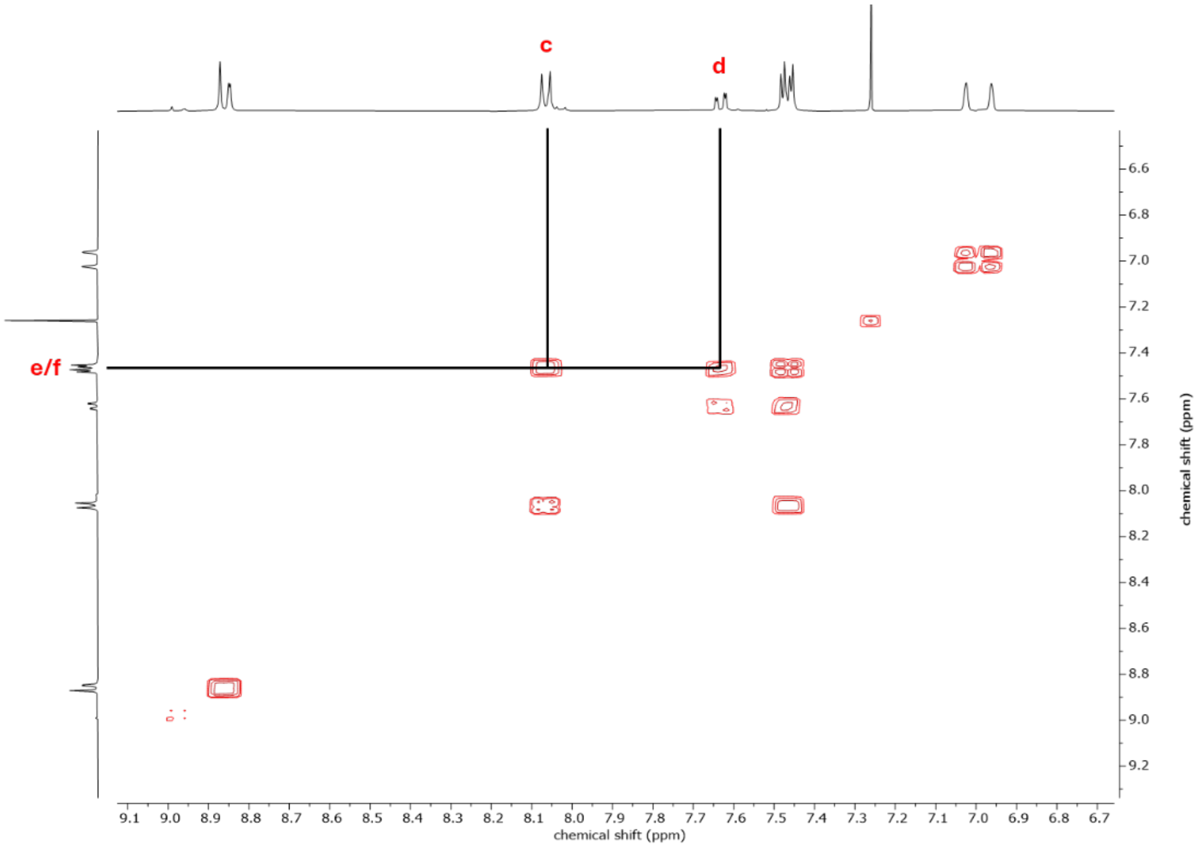

Figure S10: COSY NMR spectrum of **[^{Cl}BPIZ]Cl** in $CDCl_3$, with a drop of D_2O and $MeSO_3H$ at 25 °C.

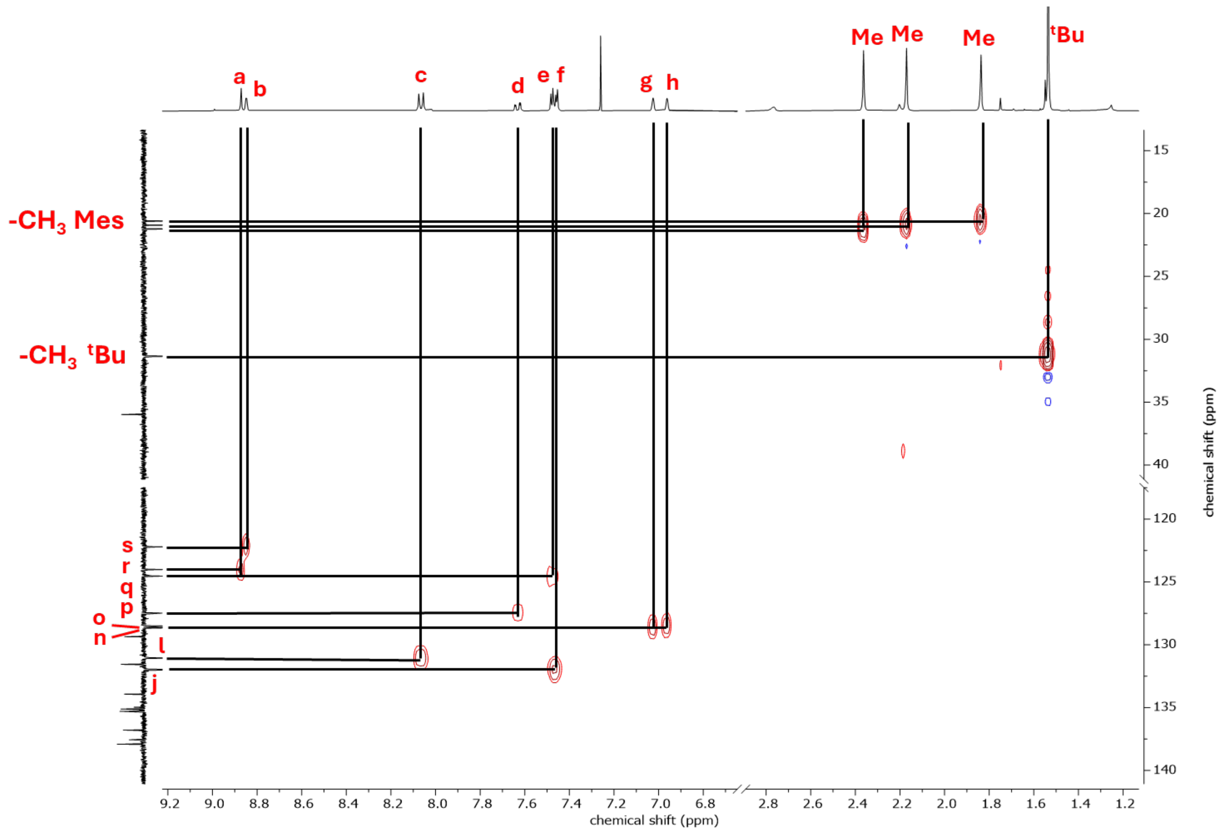

Figure S11: ^{1}H-^{13}C HSQC NMR spectrum of **[^{Cl}BPIZ]Cl** in $CDCl_3$, with a drop of D_2O and $MeSO_3H$ at 25 °C.

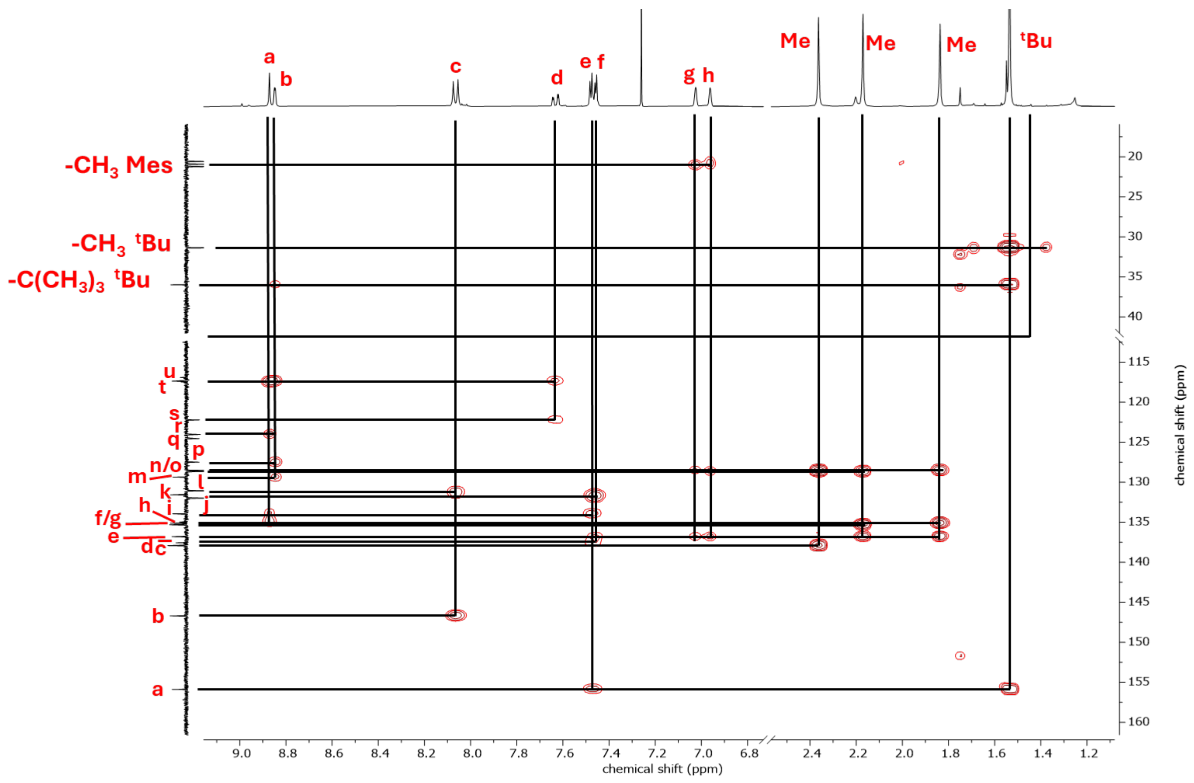

Figure S12: ^{1}H-^{13}C HMBC NMR spectrum of **[^{Cl}BPIZ]Cl** in $CDCl_3$, with a drop of D_2O and $MeSO_3H$ at 25 °C.

[^{Cl}BT-BDI-H][Sc(OTf)$_4$(MeOH)$_2$]

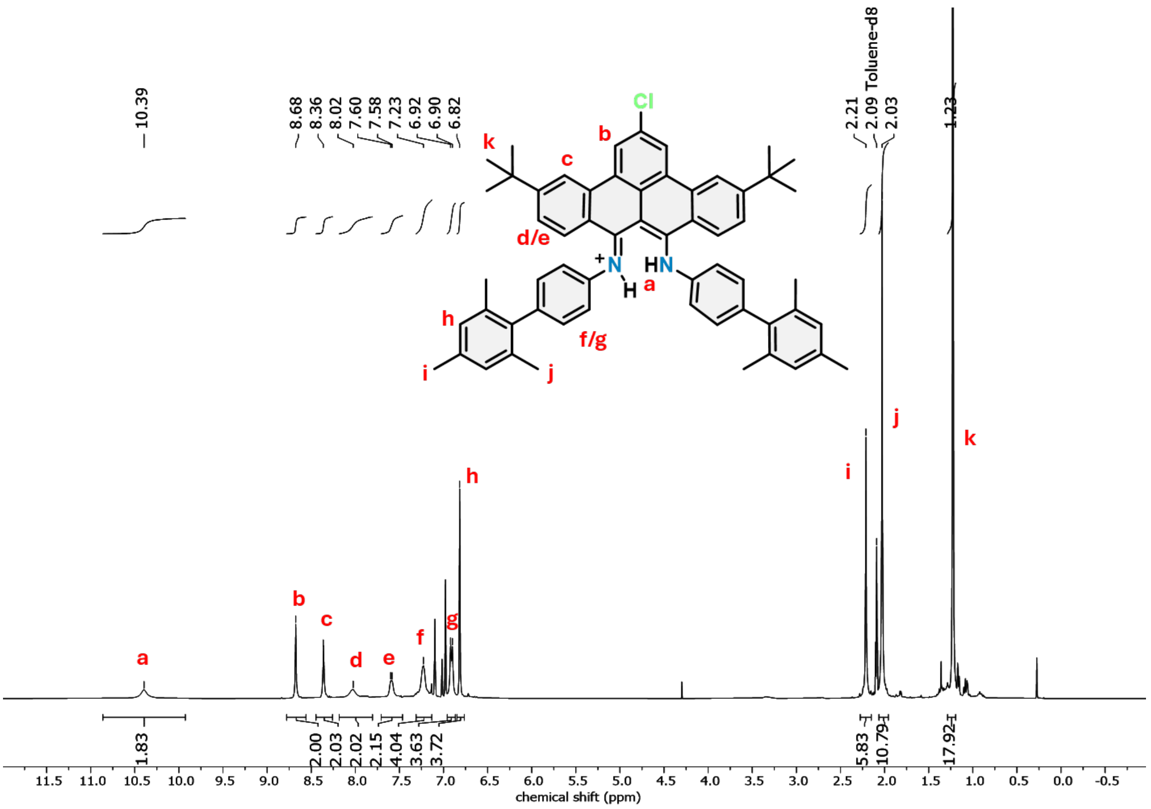

Figure S13: ^{1}H NMR spectrum of **[^{Cl}BT-BDI-H][Sc(OTf)$_4$(MeOH)$_2$]** in toluene-d_8, at 25 °C.

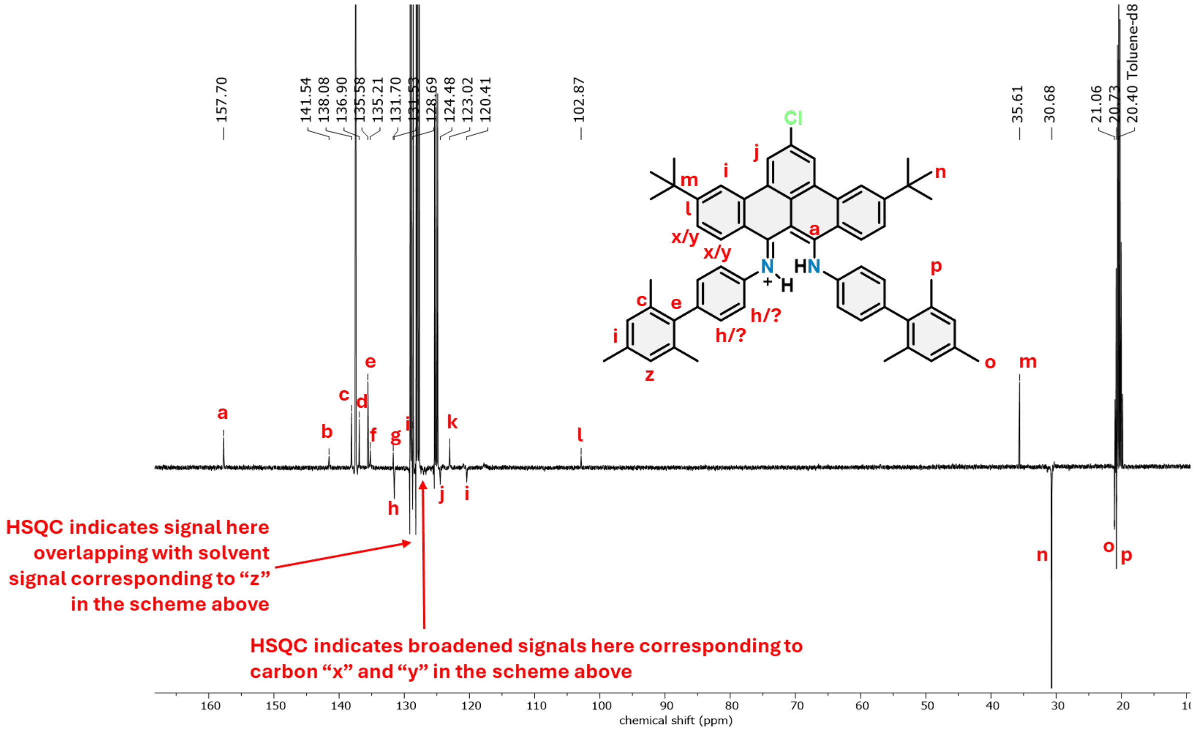

Figure S14: ^{13}C NMR spectrum of **[^{Cl}BT-BDI-H][Sc(OTf)$_4$(MeOH)$_2$]** in toluene-d_8, at 25 °C.

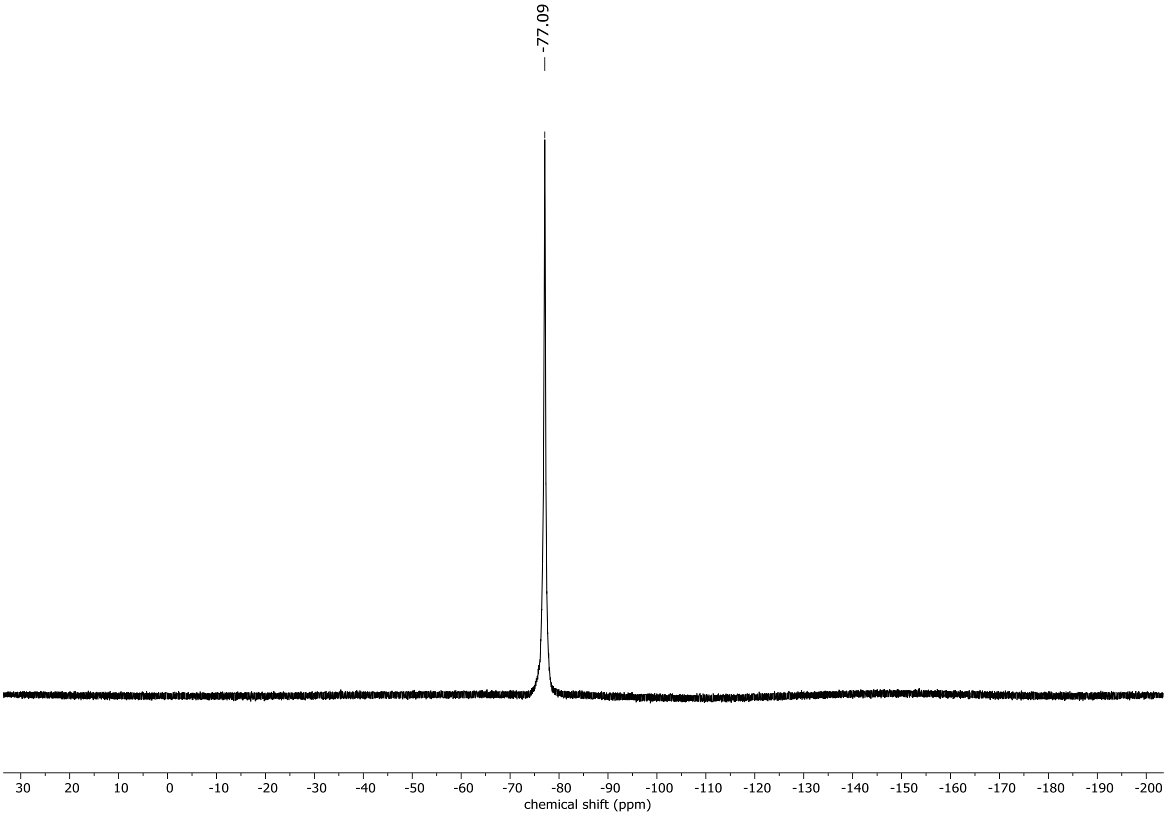

Figure S15: ^{19}F NMR spectrum of **[^{Cl}BT-BDI-H][Sc(OTf)$_4$(MeOH)$_2$]** in toluene-d_8, at 25 °C.

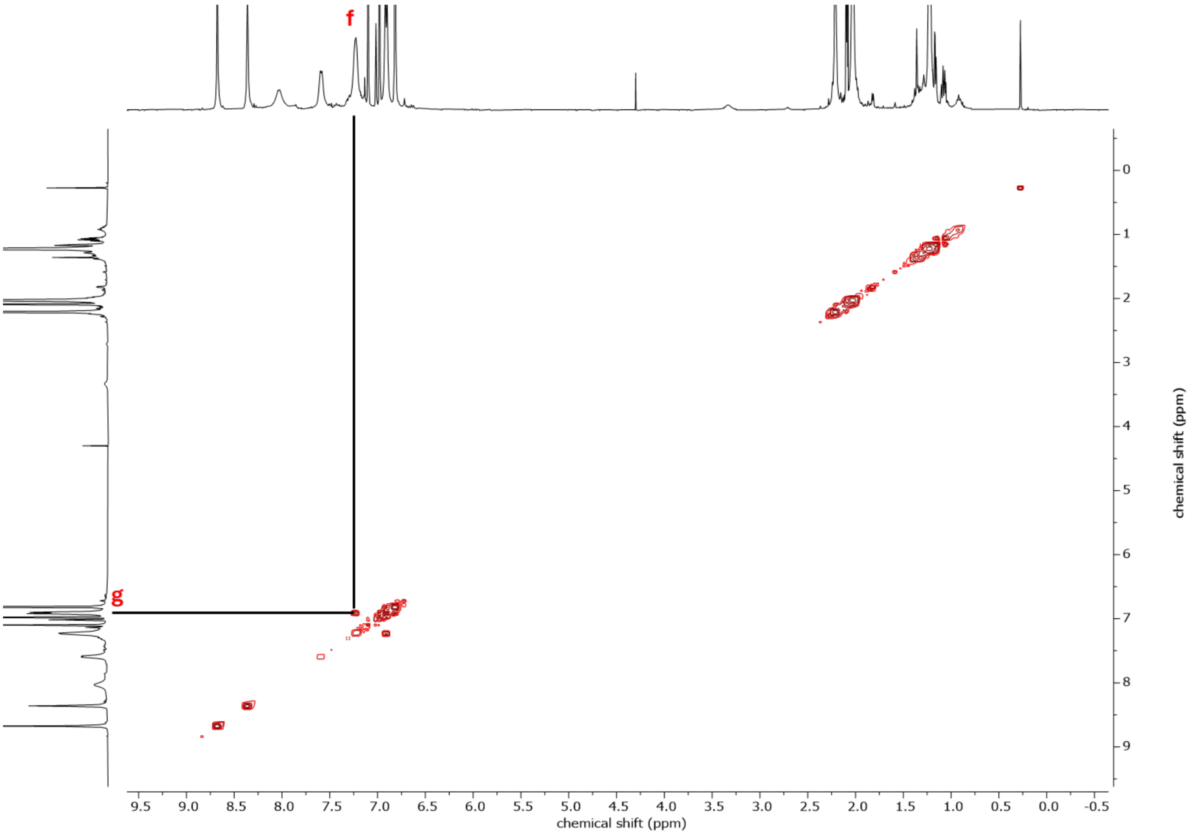

Figure S16: COSY NMR spectrum of **[^{Cl}BT-BDI-H][Sc(OTf)$_4$(MeOH)$_2$]** in toluene-d_8, at 25 °C.

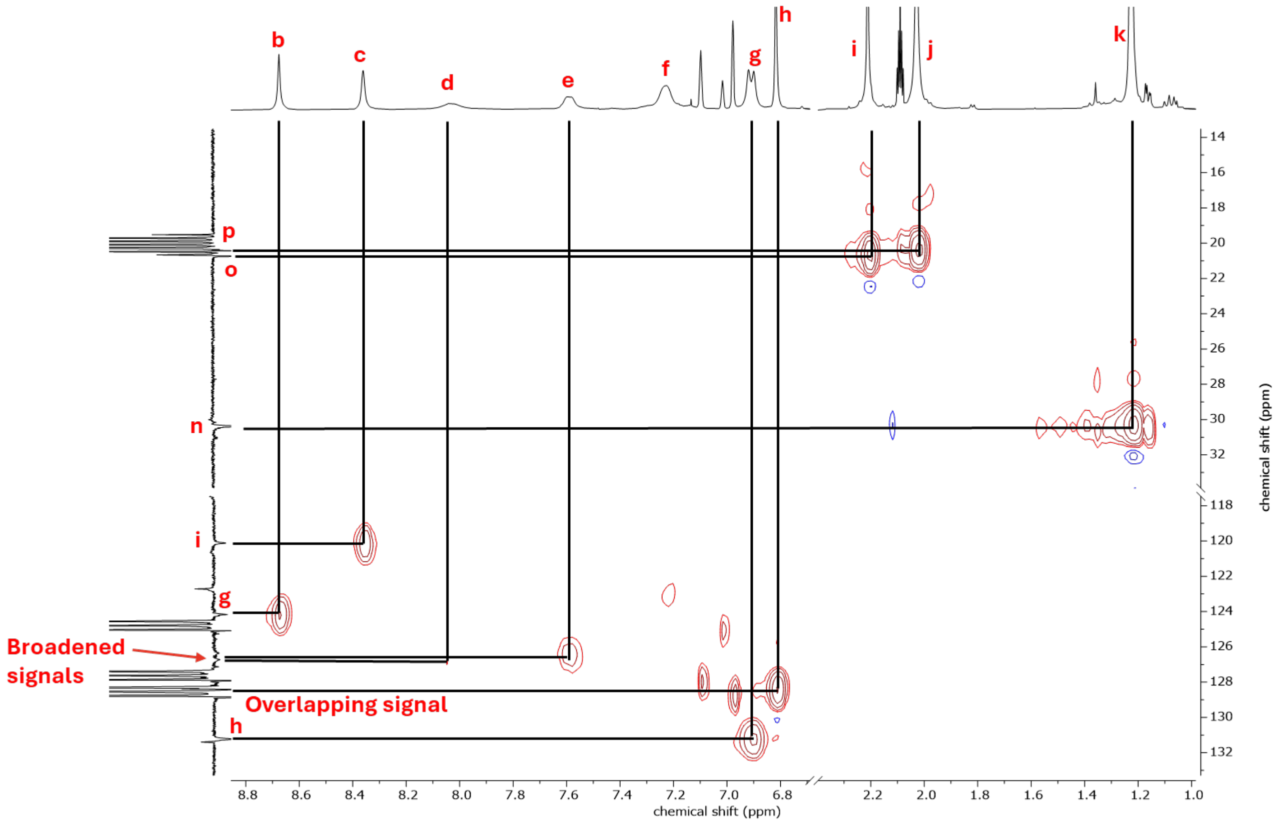

Figure S17: ^{1}H-^{13}C HSQCAD NMR spectrum of **[^{Cl}BT-BDI-H][Sc(OTf)$_4$(MeOH)$_2$]** in toluene-d_8, at 25 °C.

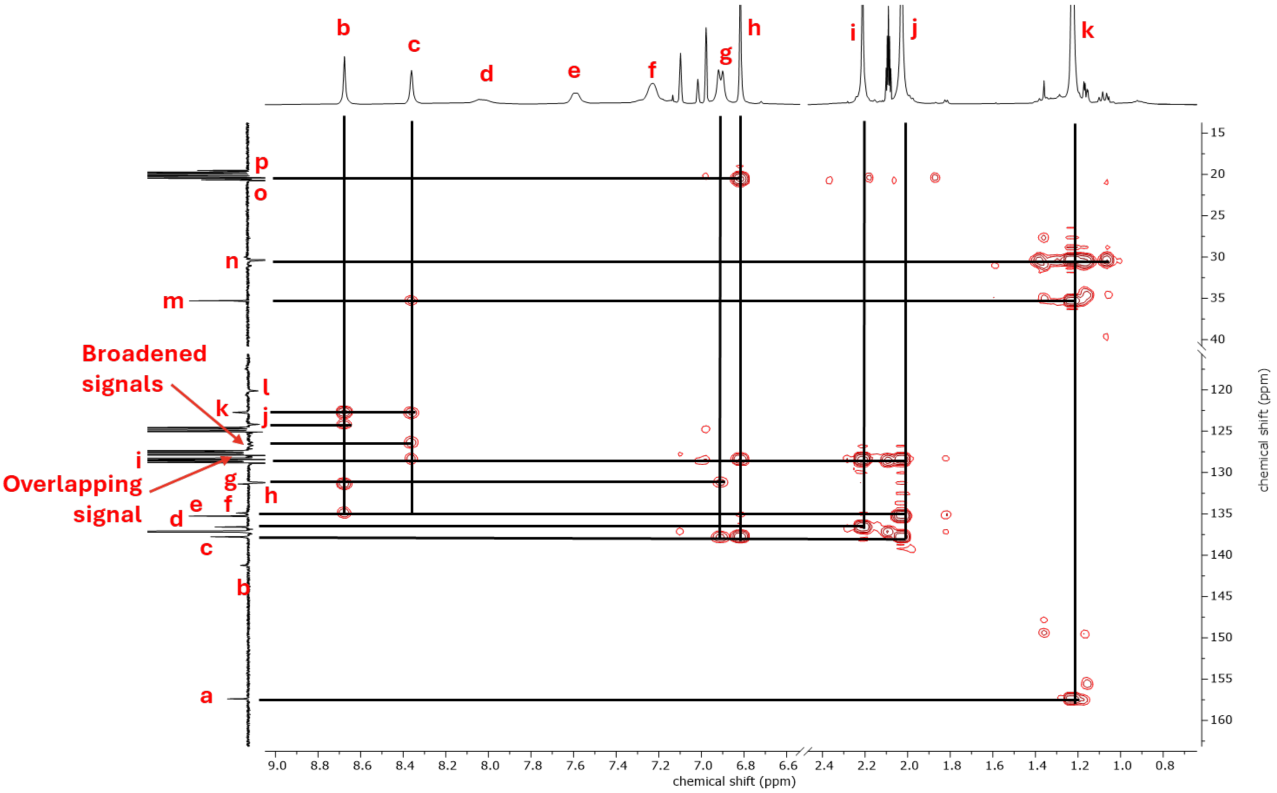

Figure S18: ^{1}H-^{13}C gHMBC NMR spectrum of **[^{Cl}BT-BDI-H][Sc(OTf)$_4$(MeOH)$_2$]** in toluene-d_8, at 25 °C.

Mechanistic investigations

Figure S19 shows the influence of $Sc(OTf)_3$ on the NMR shifts of **^{Cl}BPIZ** and **^{Cl}BBTDZ**. We hypothesize that these strong effects are due to anion exchange and coordination. The overall assignments in the NMRs below are mostly based on the observed symmetry, number of signals and integration, in addition to ESI-MS experiments to confirm the products. An overview of the different conditions leading to the different products is given in the Scheme S1:

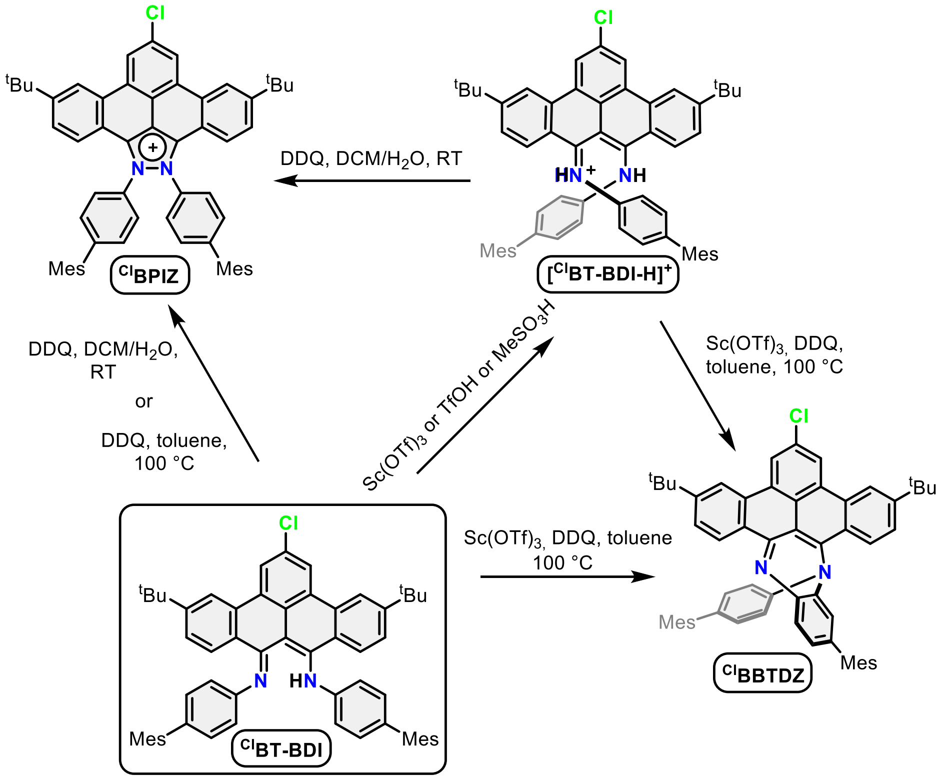

Scheme S1: An overview of the different conditions leading to different products outlined in this section.

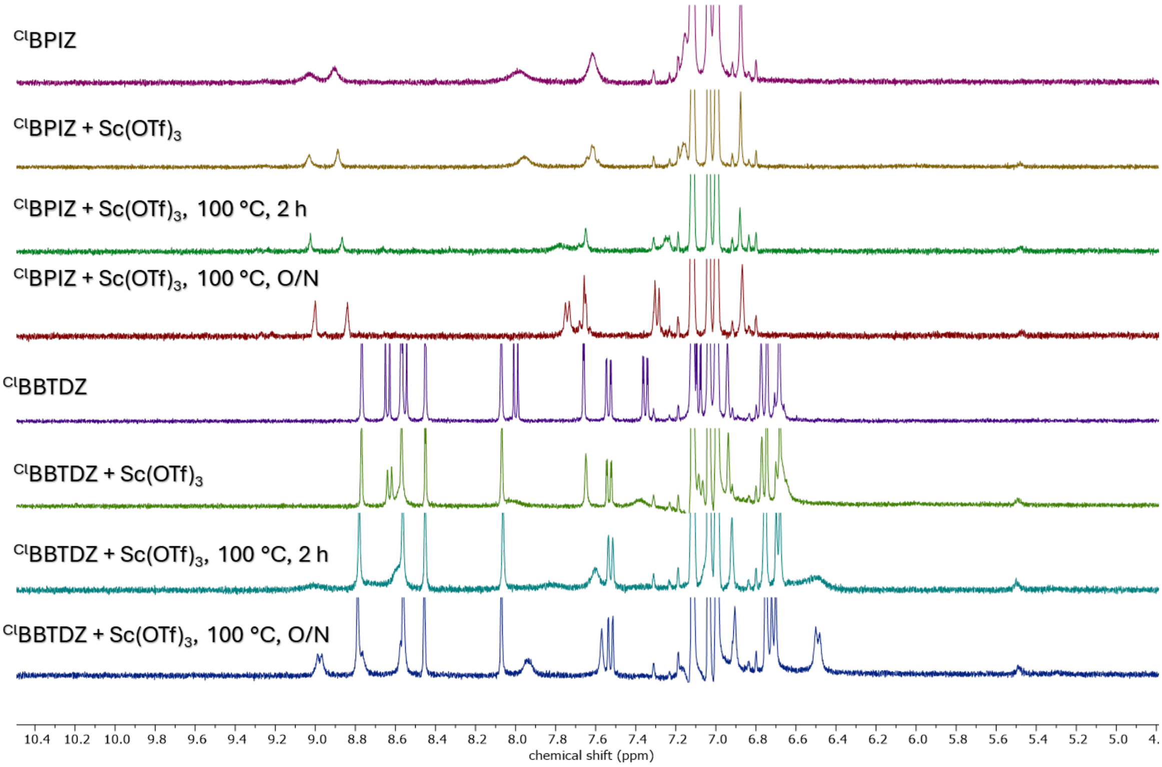

Figure S19: ^{1}H NMRs of **[^{Cl}BPIZ]Cl** (saturated) and **^{Cl}BBTDZ** (2.3 mg) with $Sc(OTf)_3$ (1.0 mg) added at different times and temperatures, showing the shift of signals under the influence of present $Sc(OTf)_3$. Spectra taken in toluene-d_8 at 25 °C. "Overnight" is abbreviated as O/N in the figure.

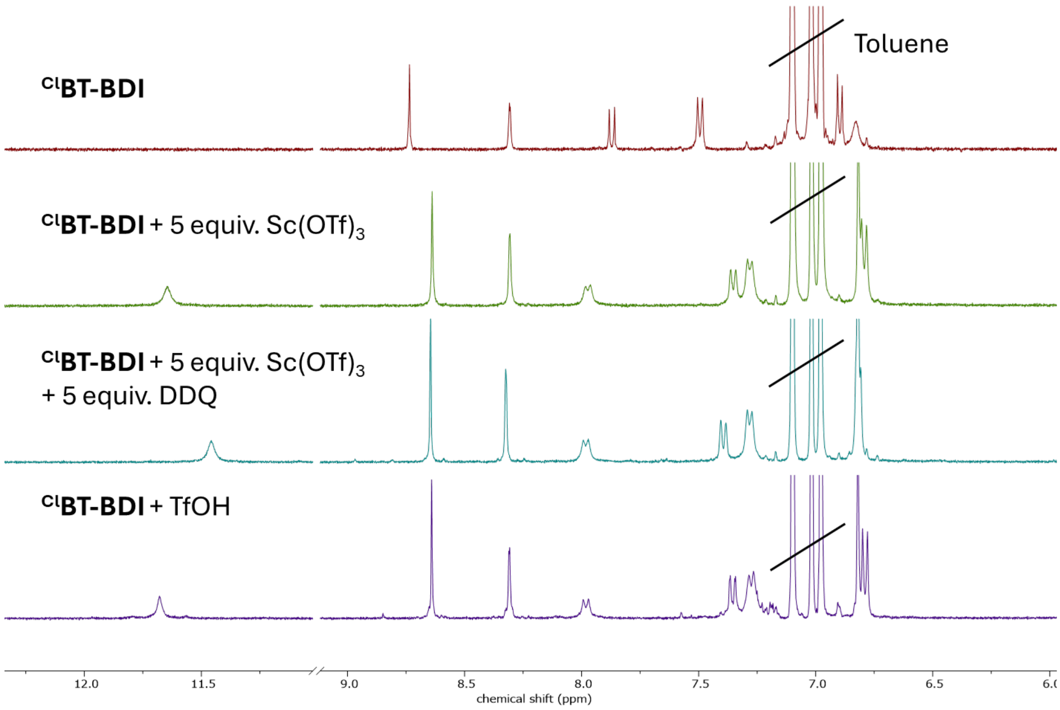

Figure S20: ^{1}H NMR spectra of **^{Cl}BT-BDI** with added $Sc(OTf)_3$, DDQ and triflic acid in toluene-d_8, at 25 °C.

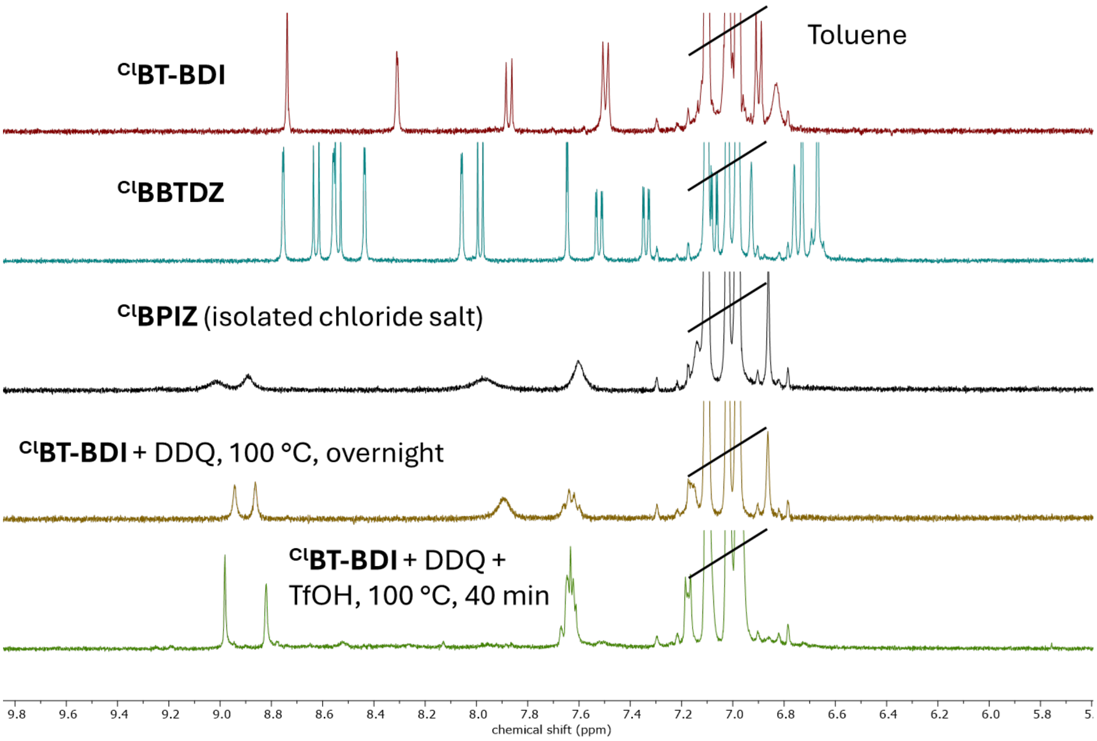

Figure S21: ^{1}H NMR spectra of **^{Cl}BT-BDI**, **^{Cl}BBTDZ** and **[^{Cl}BPIZ]Cl**, together with the spectra of the reaction of **^{Cl}BT-BDI** with DDQ and with DDQ and triflic acid at 100 °C in toluene-d_8.

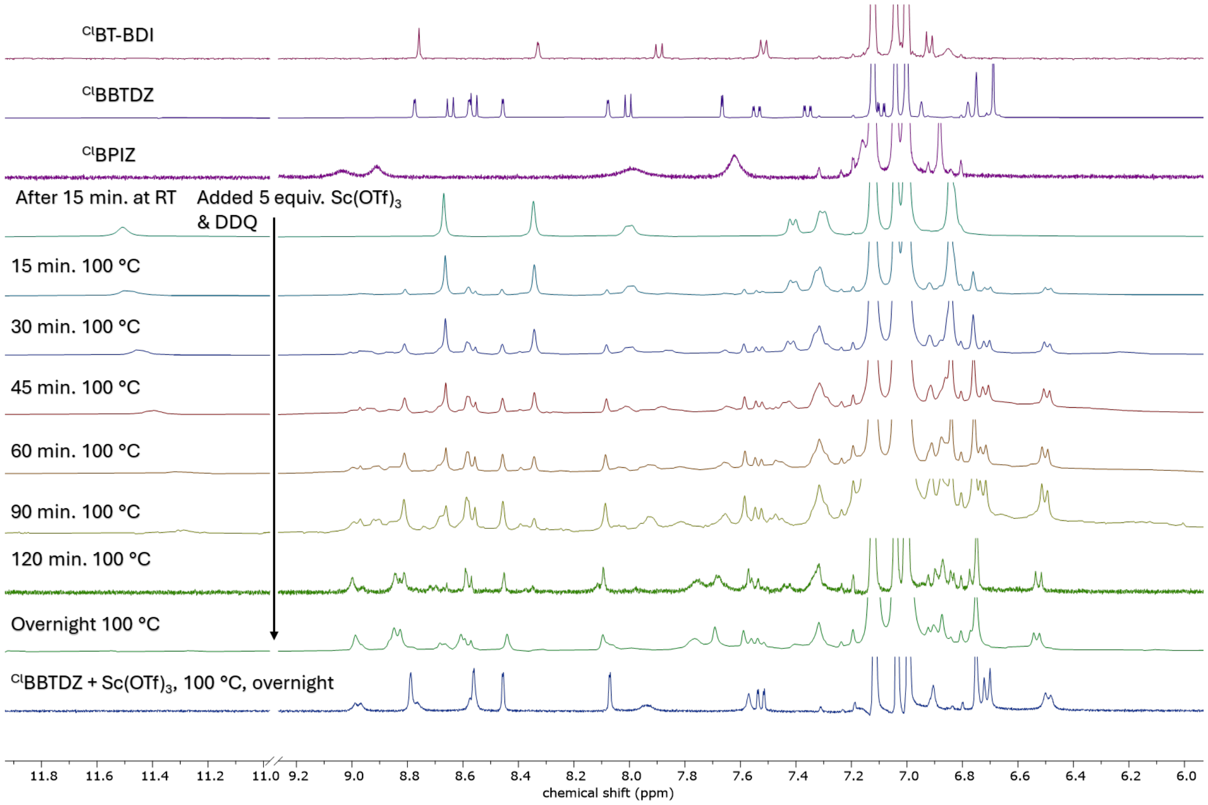

Figure S22: ^{1}H NMR spectra showing the formation of **^{Cl}BBTDZ** from ^{Cl}**BT-BDI** without **^{Cl}BPIZ** as a detectable intermediate. See also the discussion at top of this section on the influence of $Sc(OTf)_3$ on the NMR shifts.

S4 Electrochemistry

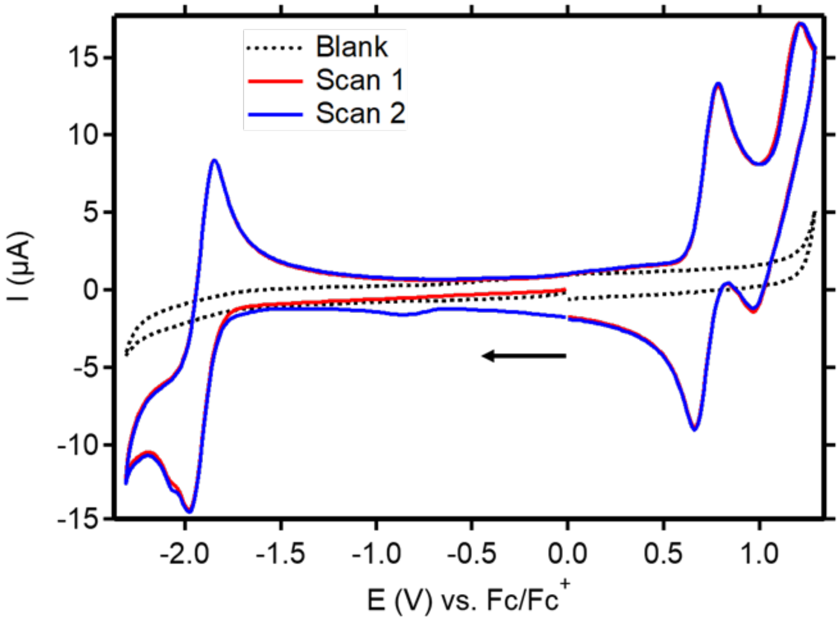

Figure S23: CV trace of ^{Cl}**BBTDZ**. Measurement was performed on 1 mM solution of analyte in DCM, 0.1 M $N^nBu_4PF_6$ using a glassy carbon WE, a Pt wire CE and a $Ag/AgNO_3$ (saturated solution in the electrolyte) RE.

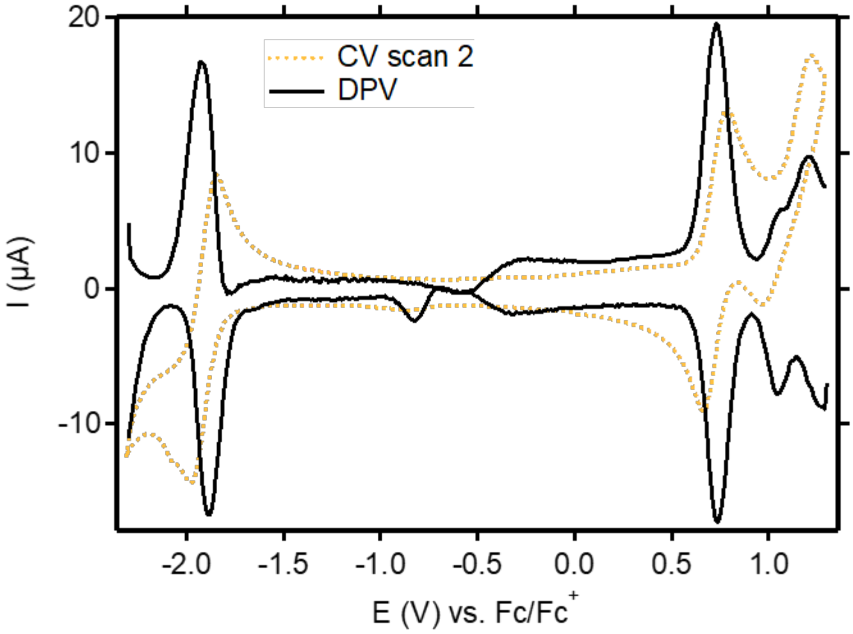

Figure S24: DPV trace of ^{Cl}**BBTDZ**. Measurement was performed on 1 mM solution of analyte in DCM, 0.1 M $N^nBu_4PF_6$ using a glassy carbon WE, a Pt wire CE and a $Ag/AgNO_3$ (saturated solution in the electrolyte) RE.

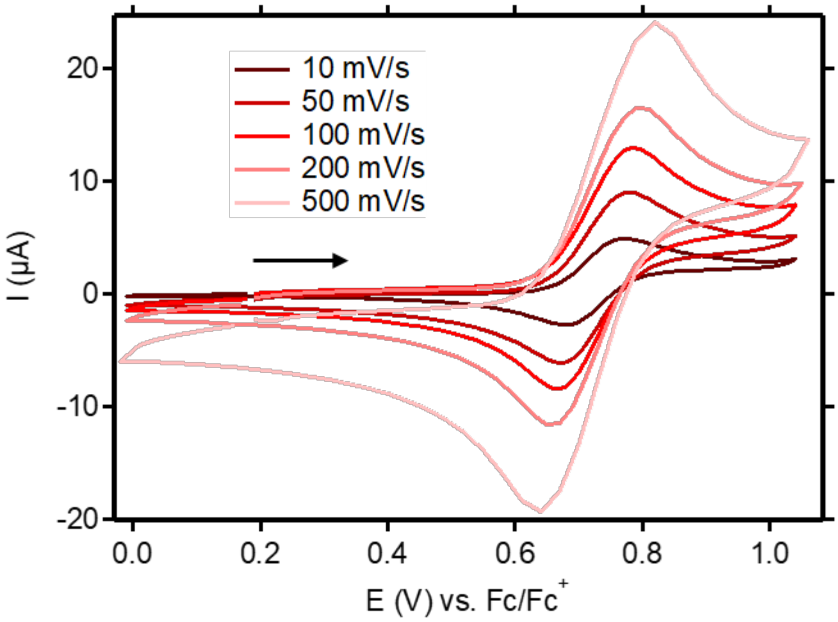

Figure S25: CV traces of the first oxidative event of ^{Cl}**BBTDZ** at various scan rates. Measurement was performed on 1 mM solution of analyte in DCM, 0.1 M $N^nBu_4PF_6$ using a glassy carbon WE, a Pt wire CE and a $Ag/AgNO_3$ (saturated solution in the electrolyte) RE.

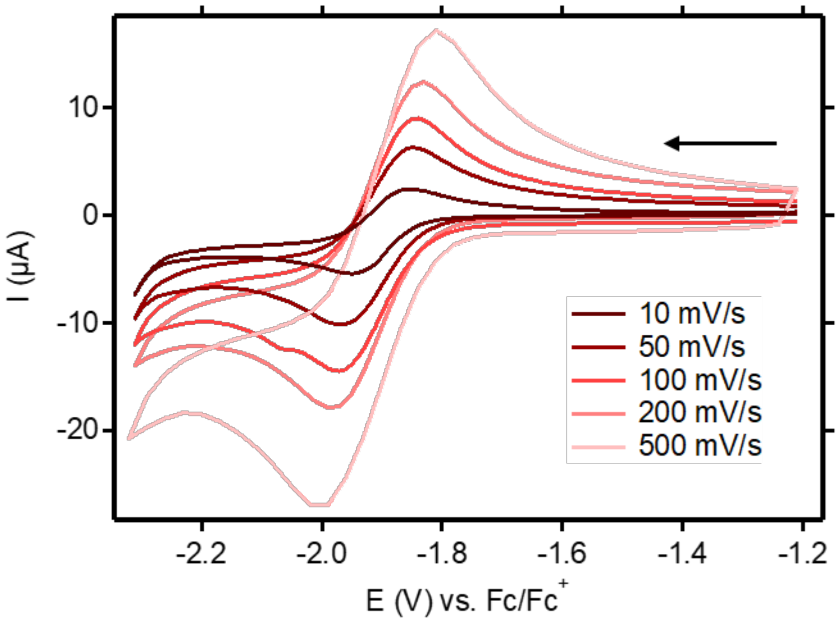

Figure S26: CV traces of the first reductive event of ^{Cl}**BBTDZ** at various scan rates. Measurement was performed on 1 mM solution of analyte in DCM, 0.1 M $N^nBu_4PF_6$ using a glassy carbon WE, a Pt wire CE and a $Ag/AgNO_3$ (saturated solution in the electrolyte) RE.

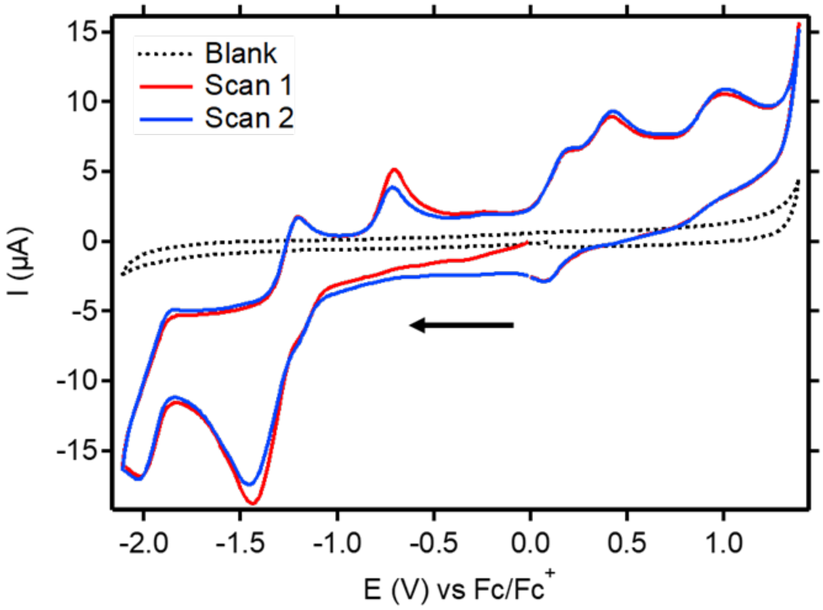

Figure S27: CV trace of **[^{Cl}BPIZ]Cl**. Measurement was performed on 1 mM solution of analyte in DCM, 0.1 M $N^nBu_4PF_6$ using a glassy carbon WE, a Pt wire CE and a $Ag/AgNO_3$(saturated solution in the electrolyte) RE.

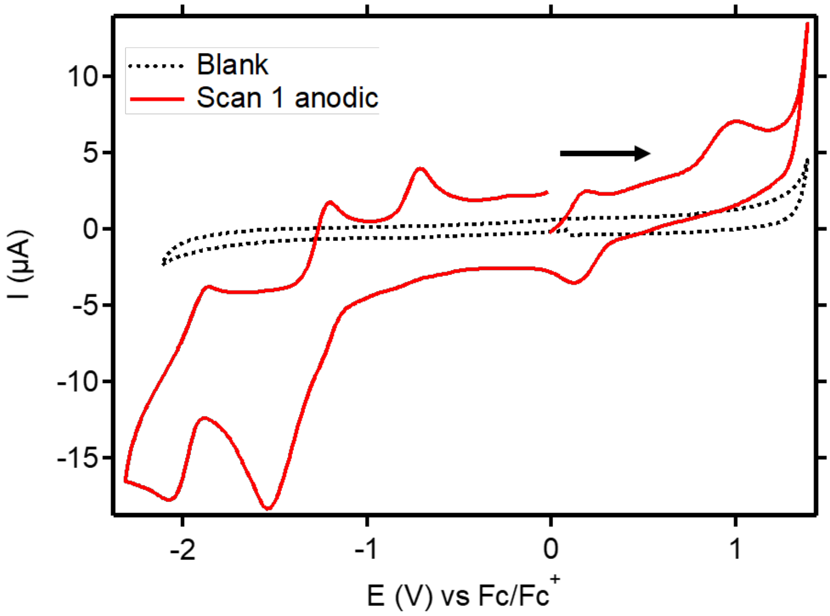

Figure S28: CV trace of **[^{Cl}BPIZ]Cl**. Measurement was performed on 1 mM solution of analyte in DCM, 0.1 M $N^nBu_4PF_6$ using a glassy carbon WE, a Pt wire CE and a $Ag/AgNO_3$ (saturated solution in the electrolyte) RE.

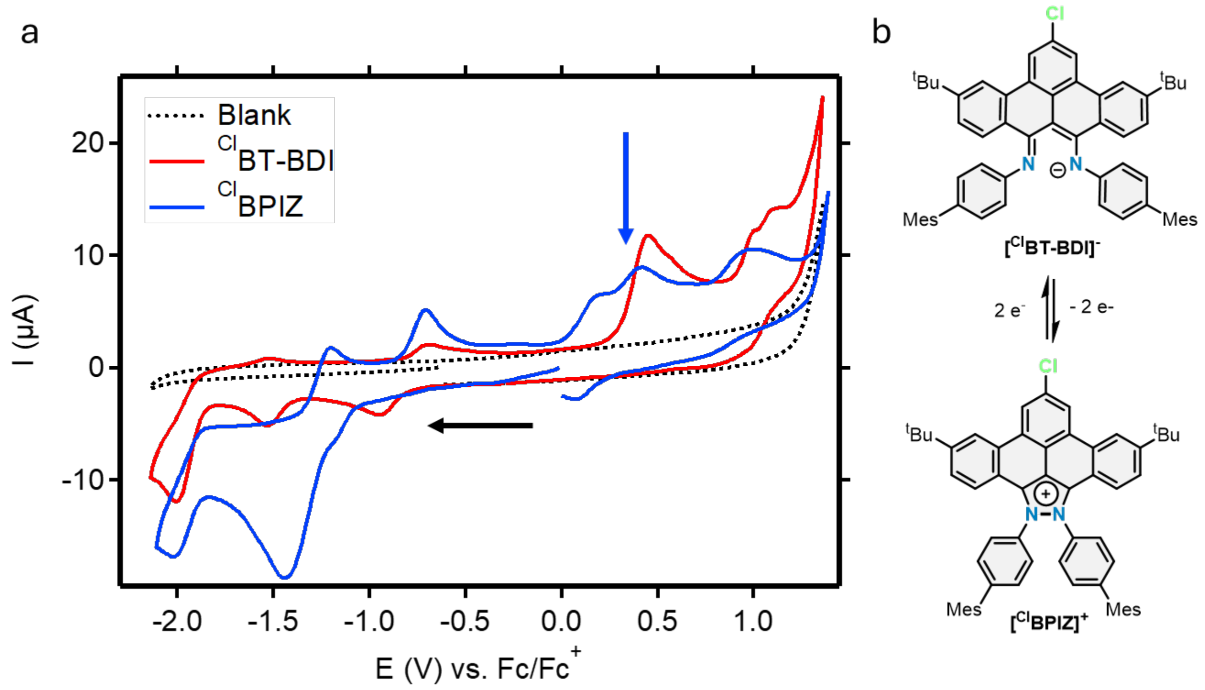

Figure S29: a) CV trace of **[^{Cl}BPIZ]Cl** and **^{Cl}BT-BDI** (second scan).[1] The blue arrow indicates the oxidation event in the backward (anodic) scan of **^{Cl}BPIZ** that is indicative of the formation of **^{Cl}BT-BDI** in the forward (cathodic) scan. Measurement was performed on 1 mM solution of analyte in DCM, 0.1 M $N^nBu_4PF_6$ using a glassy carbon WE, a Pt wire CE and a $Ag/AgNO_3$ (saturated solution in the electrolyte) RE. b) Scheme showing the interconversion of **[^{Cl}BT-BDI]$^-$** and **[^{Cl}BPIZ]$^+$**.

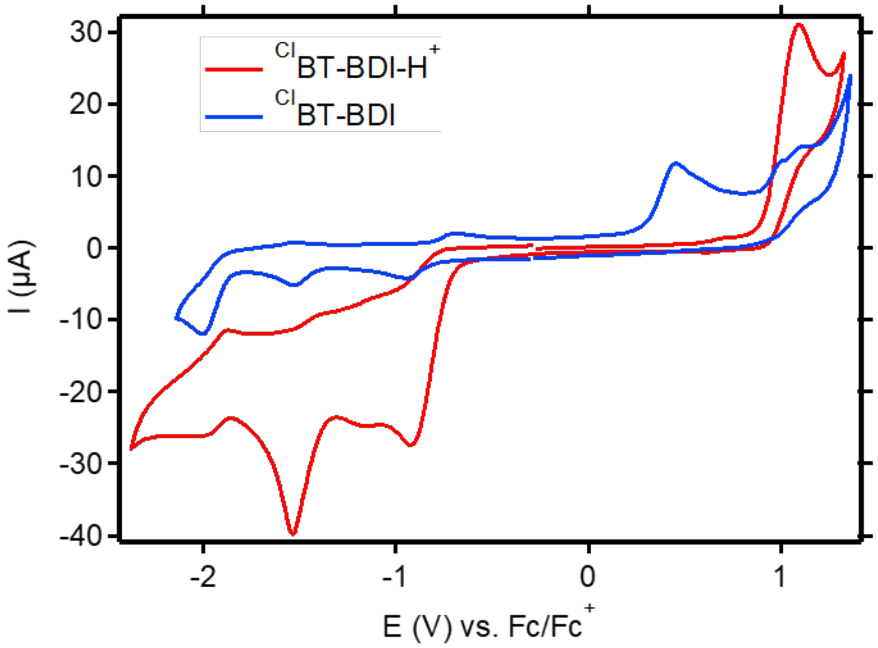

Figure S30: CV trace of **[^{Cl}BT-BDI-H][Sc(OTf)$_4$(MeOH)$_2$]** (2 mM) compared to that of **^{Cl}BT-BDI** (1 mM). Measurement was performed in DCM, 0.1 M $N^nBu_4PF_6$ using a glassy carbon WE, a Pt wire CE and a $Ag/AgNO_3$ (saturated solution in the electrolyte) RE.

S5 UV-Vis and PL spectroscopy

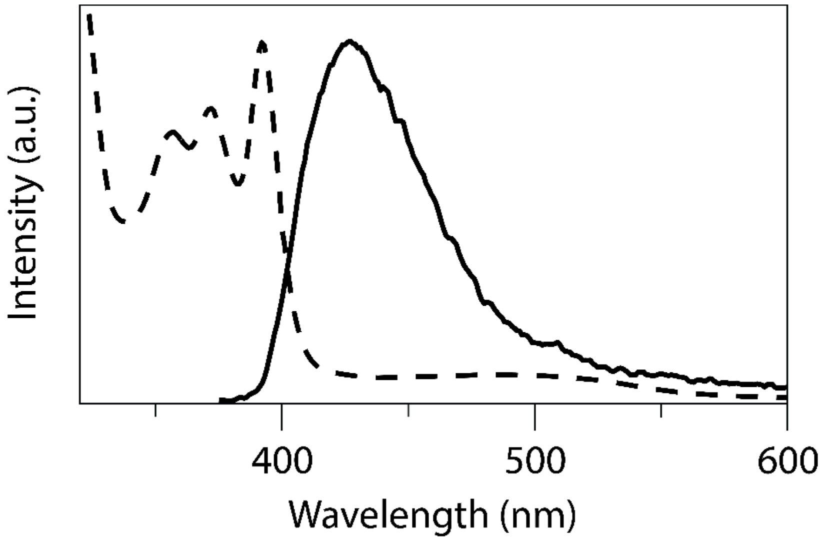

Figure S31: Absorption (dotted line) and emission (solid line, excitation at 350 nm) of **[^{Cl}BPIZ]Cl** in DCM (25 µM for the absorption, 50 µM for the emission).

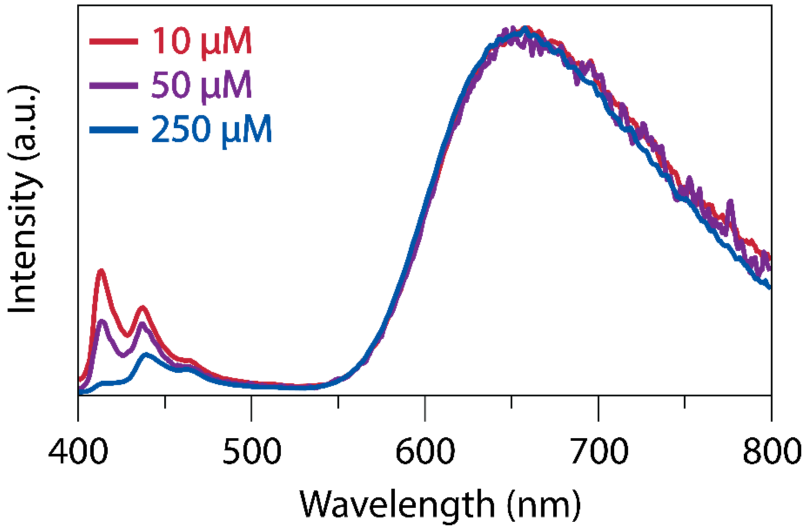

Figure S32: Emission spectra of **^{Cl}BBTDZ** at concentrations of 10, 50 and 250 µM in DCM. Excitation at 350 nm. The intensity of the blue emission decreases at lower concentrations, most likely due to reabsorption of the zero-phonon line at 413 nm.

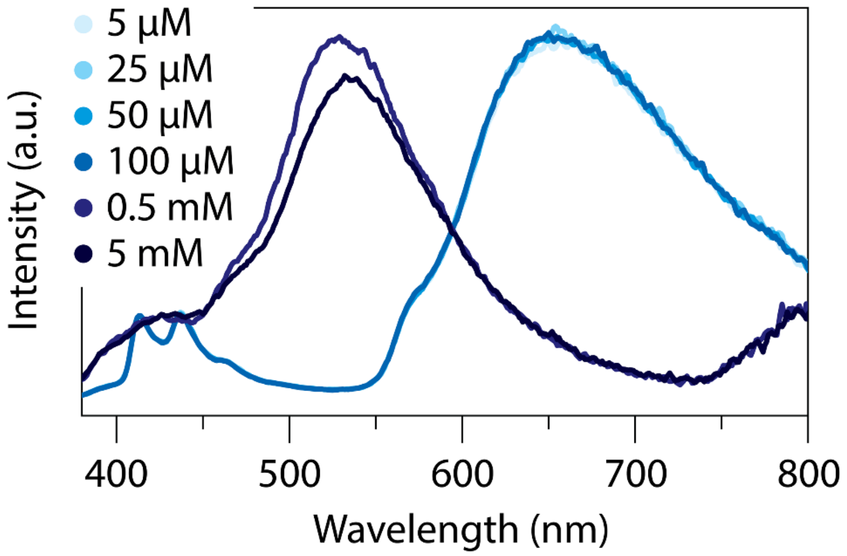

Figure S33: Emission spectra of ^{Cl}**BBTDZ** (50 µM) in DCM with a range of methanesulfonic acid concentrations. Excitation at 350 nm. For high concentrations of methanesulfonic acid (10 and 100 equivalents), likely protonation of ^{Cl}**BBTDZ** and changes to the polarity of the medium lead to the observed changes in the emission. No changes are seen upon the addition of 0.1, 0.5, 1 or 2 equivalents of methanesulfonic acid. This shows that acid-base equilibria are not involved in the dual emission of ^{Cl}**BBTDZ** under the standard conditions.

S6 X-ray crystal structure determinations

CCDC 2441497-2441498 contain the supplementary crystallographic data for this paper. These data can be obtained free of charge from The Cambridge Crystallographic Data Centre *via* www.ccdc.cam.ac.uk/data_request/cif.

^{Cl}BBTDZ

$C_{59}H_{55}ClN_2$ + disordered solvent, Fw = 827.50*, yellow needle, 0.47 × 0.07 × 0.05 mm^3, triclinic, $P\overline{1}$ (no. 2), a = 14.0705(7), b = 14.3562(8), c = 15.4919(9) Å, α = 89.966(3), β = 65.879(2), γ = 61.253(1) °, V = 2427.8(2) Å^3, Z = 2, D_x = 1.132 g/cm^3*, μ = 0.12 mm^{-1}*. The diffraction experiment was performed on a Bruker Kappa ApexII diffractometer with sealed tube and Triumph monochromator (λ = 0.71073 Å) at a temperature of 150(2) K up to a resolution of $(\sin \theta/\lambda)_{max}$ = 0.61 Å^{-1}. The crystal was cracked into two fragments related by a 5.8° rotation about an arbitrary axis. Consequently, two orientation matrices were used for the intensity integration with the Eval15 software.[25] A multi-scan absorption correction and scaling was performed with TWINAB[26] (correction range 0.62-0.75). A total of 39257 reflections was measured, 9045 reflections were unique (R_{int} = 0.070), 5393 reflections were observed [$I>2\sigma(I)$]. The structure was solved with Patterson superposition methods using SHELXT.[27] Structure refinement was performed with SHELXL-2019[28] on F^2 of all reflections. The crystal structure contains large voids (228 Å^3 / unit cell) filled with disordered CH_2Cl_2 molecules. Their contribution to the structure factors was secured by back-Fourier transformation using the SQUEEZE algorithm[29] resulting in 80 electrons / unit cell. Non-hydrogen atoms were refined freely with anisotropic displacement parameters. Hydrogen atoms were introduced in calculated positions and refined with a riding model. 573 Parameters were refined with no restraints. R1/wR2 [$I > 2\sigma(I)$]: 0.0595 / 0.1327. R1/wR2 [all refl.]: 0.1111 / 0.1544. S = 1.033. Residual electron density between -0.24 and 0.29 e/Å^3. Geometry calculations and checking for higher symmetry was performed with the PLATON program.[30]

*Derived values do not contain the contribution of the disordered solvent molecules.

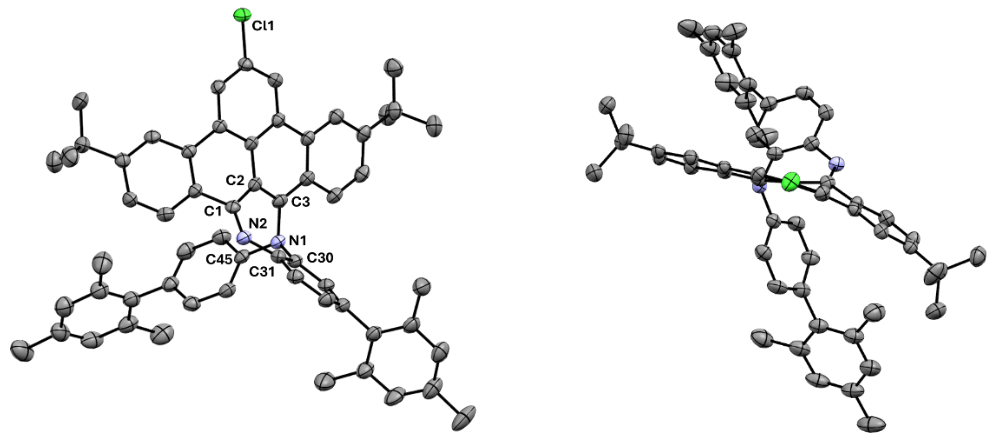

Figure S34: Displacement ellipsoid plot (50 % probability) of the asymmetric unit of ^{Cl}**BBTDZ** seen from two directions. Hydrogen atoms and severely disordered CH_2Cl_2 solvent molecules are omitted for clarity.

Table S1: Selected bond distances and angles in the crystal structure of ^{Cl}**BBTDZ**.

Bond	Distance (Å) or angle (°)
**N1 – C3**	1.445(3)
**N1 – C30**	1.439(3)
**N2 – C1**	1.299(3)
**N2 – C31**	1.401(3)
**C1 – C2**	1.462(4)
**C2 – C3**	1.367(4)
**C30 – C31**	1.398(4)
**C3 – N1 – C30**	109.3(2)
**C3 – N1 – C45**	116.0(2)
**C30 – N1 – C45**	116.3(2)

[^{Cl}BT-BDI-H][Sc(OTf)$_4$(MeOH)$_2$]

[$C_{59}H_{58}ClN_2$]($C_6H_8F_{12}O_{14}Sc$)·4$C_5H_{12}O$ + disordered solvent, Fw = 1888.43*, dark green block, 0.43 × 0.41 × 0.32 mm^3, monoclinic, I2/a (no. 15), a = 21.2165(5), b = 24.4899(6), c = 21.6136(4) Å, β = 105.483(1) °, V = 10822.7(4) Å^3, Z = 4, D_x = 1.159 g/cm^3*, μ = 0.25 mm^{-1}*. The diffraction experiment was performed on a Bruker Kappa ApexII diffractometer with sealed tube and Triumph monochromator (λ = 0.71073 Å) at a temperature of 150(2) K up to a resolution of $(\sin \theta/\lambda)_{max}$ = 0.65 Å^{-1}. The diffraction pattern contains a significant amount of diffuse scattering. Intensity integration was performed with the Eval15 software.[25] A multi-scan absorption correction and scaling was performed with SADABS[31] (correction range 0.60-0.75). A total of 133493 reflections was measured, 12445 reflections were unique (R_{int} = 0.041), 7431 reflections were observed [$I>2\sigma(I)$]. The structure was solved with Patterson superposition methods using SHELXT.[27] Structure refinement was performed with SHELXL-2019[28] on F^2 of all reflections. The *tert*-butyl group of the cation and the triflate ligands of the anion were refined with disorder models. In addition to the MTBE molecules in the atomic structure model, severely disordered MTBE molecules were handled with the SQUEEZE algorithm[29] resulting in 381 electrons / unit cell in voids of 1809 Å^3 / unit cell. Non-hydrogen atoms were refined freely with anisotropic displacement parameters. Hydrogen atoms in the ordered part were located in difference Fourier maps, hydrogen atoms in the disordered parts were introduced in calculated positions. All hydrogen atoms were refined with a riding model. 741 Parameters were refined with 1096 restraints (geometries and displacement parameters in the disordered groups and in the modelled MTBE molecules). R1/wR2 [$I > 2\sigma(I)$]: 0.0674 / 0.2092. R1/wR2 [all refl.]: 0.1011 / 0.2429. S = 1.046. Residual electron density between -0.63 and 0.63 e/Å^3. Geometry calculations and checking for higher symmetry was performed with the PLATON program.[30]

*Derived values do not contain the contribution of the disordered solvent molecules.

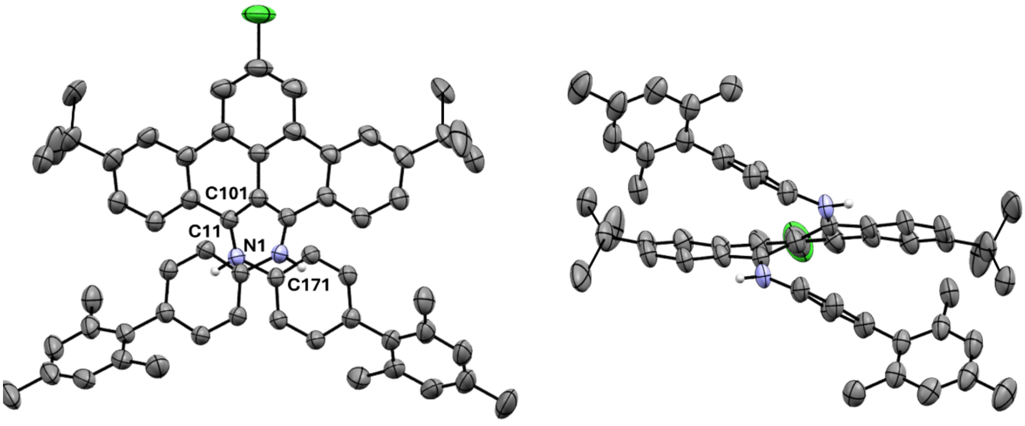

Figure S35: Displacement ellipsoid plot (50 % probability) of **[^{Cl}BT-BDI-H][Sc(OTf)$_4$(MeOH)$_2$]** seen from two directions. Hydrogen atoms – except for those bonded to N1 – **[Sc(OTf)$_4$(MeOH)$_2$]$^-$** counterion and MTBE solvent molecules are omitted for clarity. Only the major disorder component is shown for the *tert*-butyl group.

Table S2: Selected bond distances in the crystal structure of **[^{Cl}BT-BDI-H][Sc(OTf)$_4$(MeOH)$_2$]**.

Bond	Distance (Å)
**N1 – C11**	1.339(3)
**N1 – C171**	1.437(3)
**C11 – C101**	1.416(2)

S7 IR spectra

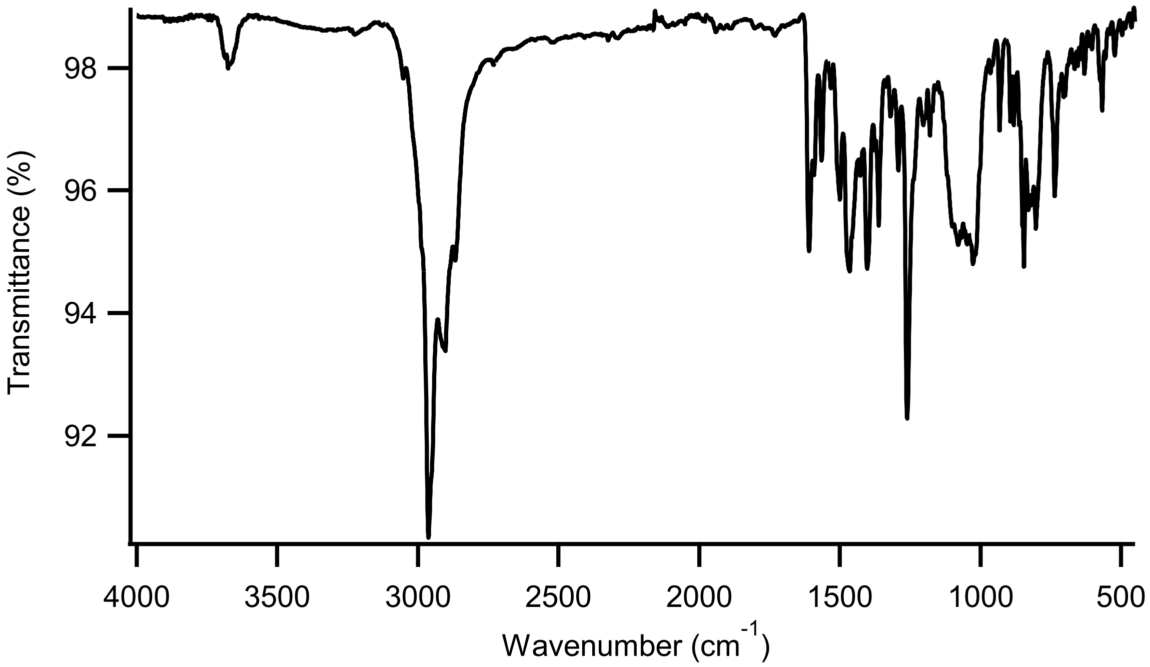

Figure S36: ATR-IR spectrum of **^{Cl}BBTDZ**.

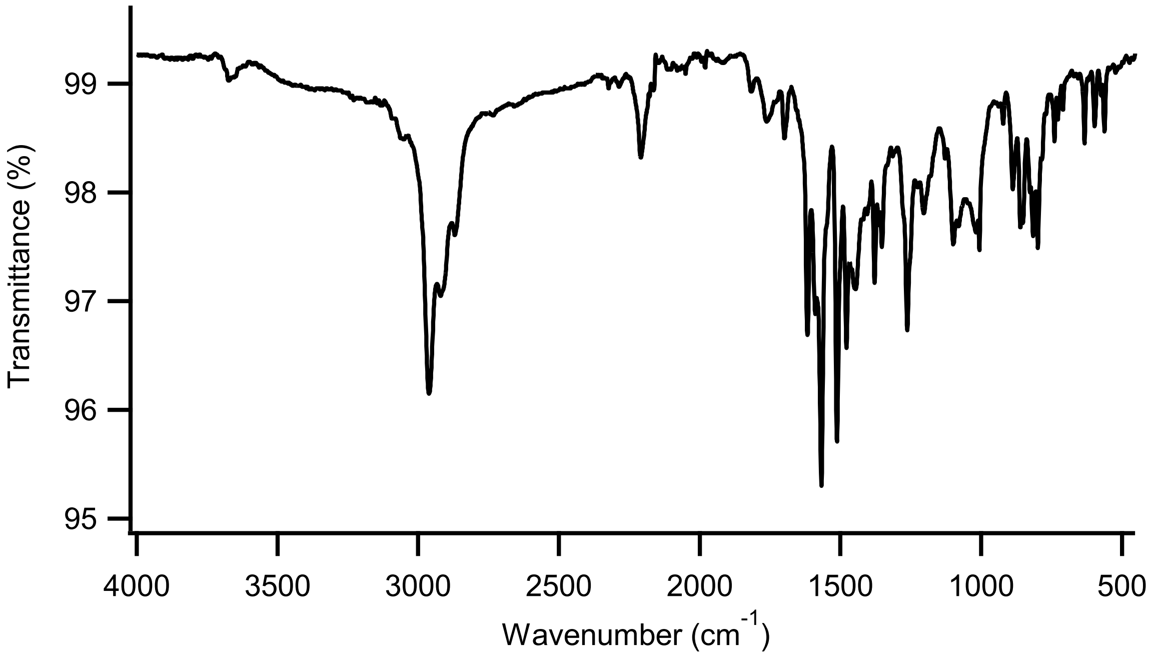

Figure S37: ATR-IR spectrum of **[^{Cl}BPIZ]Cl**. The signal at 2208 cm^{-1} is attributed to nitrile stretch vibrations from residual DDQ.

S8 HRMS spectra

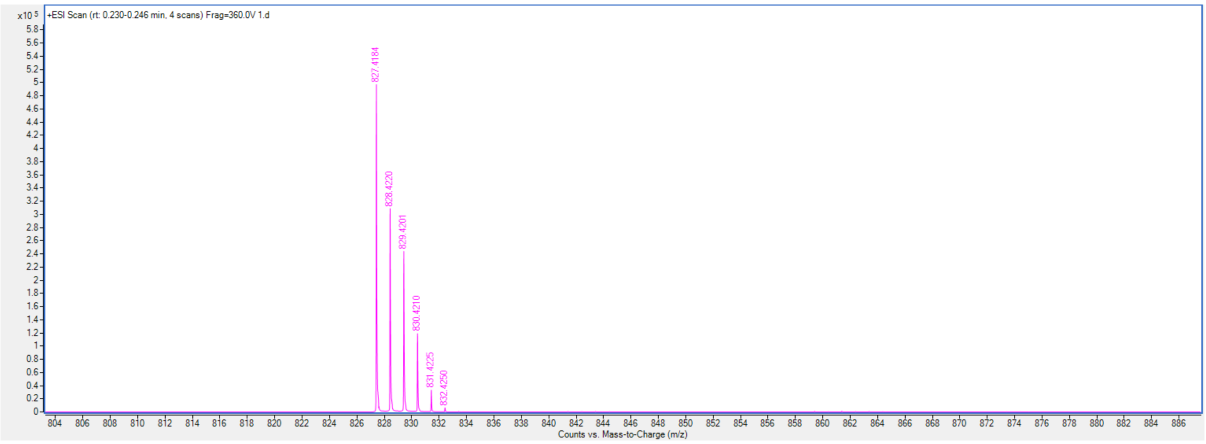

Figure S38: HRMS spectrum of ^{Cl}**BBTDZ** {m/z = 827.4184 [M+H]$^+$, calc. 827.4132}.

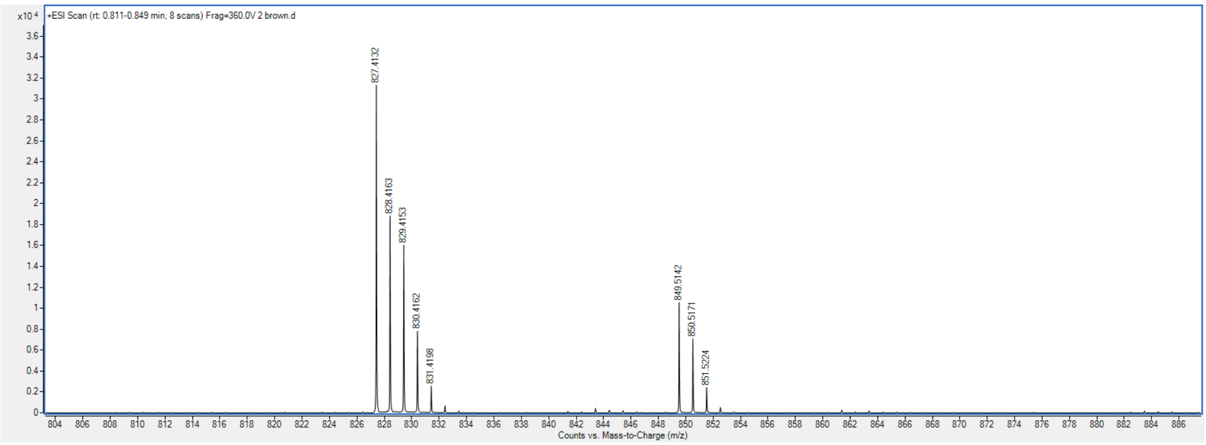

Figure S39: HRMS spectrum of ^{Cl}**BPIZ** {m/z = 827.4132 [M]$^+$, calc. 827.4132}. The signal at m/z = 849.5142 corresponds to the calculated mass of the analogous *tert*-butyl substituted compound {[M]$^+$, calc. 849.5148}, derived from ^{tBu}**BT-BDI**[1] which is present as a minor impurity based on ^{1}H NMR spectroscopy (*vide supra*).

S9 Computational results

The input, log and formatted checkpoint files for S_0, S_1, S_2, S_3, S_4 and S_5 states can be accessed in the Yoda data repository at https://doi.org/10.24416/UU01-5PNSDH. Table S3 shows the TD-DFT results for the S_1 state discussed in the manuscript.

The data in Table S3 raises some additional questions. Unexpectedly, the first excited state shows a HOMO → LUMO transition with an excitation energy wavelength of 743.46 nm with a low oscillator strength of 0.0134. Contrary to our expectations, a screening of various long-range corrected functionals and more extensive basis sets did not eliminate this "dark" excitation and gave inconsistent results, highlighting the complex nature of this mechanism. More elaborate Post-Hartree-Fock methods such as Møller-Plesset perturbation and/or Coupled Cluster theory were not considered due to their high computational cost for asymmetric molecules of our scale. Truncated model compounds of ^{Cl}**BBTDZ** did not give any reasonable excitation profiles, facilitating the need of including each component of the molecule to simulate the observables. While the HOMO–LUMO energy gap — rather than the HOMO → LUMO excitation energy — exhibits reasonable agreement with experimental spectroscopic and electrochemical data for both the ground state and the optimized S_1 state, this agreement must be taken as a crude approximation. We are therefore cautious to over-interpret the data. Nevertheless, the computational results presented here and in the main text provide a valuable qualitative perspective on the excited-state structure and associated energetics.

Table S3: Parameters of first five excitations of ^{Cl}**BBTDZ** (B3LYP, 6-311+G(d,p), optimized for first excitation).

Excited state	Electronic transition	Orbital composition	Configuration-Interaction	Excitation energy / eV (nm)	Oscillator strength *f*
1	S_0-S_1	HOMO→LUMO	0.70143	1.6677 (743.46)	0.0134
2	S_0-S_2	HOMO-1→LUMO	0.69951	2.4807 (499.79)	0.4255
3	S_0-S_9<br>S_0-S_3<br>S_0-S_2	HOMO-8→LUMO<br>HOMO-2→LUMO<br>HOMO→LUMO+1	-0.12223<br>0.56141<br>-0.36040	2.8983 (427.78)	0.2969
4	S_0-S_3<br>S_0-S_2	HOMO-2→LUMO<br>HOMO→LUMO+1	0.35643<br>0.58239	2.9218 (424.34)	0.1035
5	S_0-S_8<br>S_0-S_6<br>S_0-S_4<br>S_0-S_3<br>S_0-S_2	HOMO-7→LUMO<br>HOMO-5→LUMO<br>HOMO-3→LUMO<br>HOMO-1→LUMO+1<br>HOMO→LUMO+1	0.20128<br>0.56522<br>0.29320<br>0.14370<br>-0.12908	3.0984 (400.15)	0.0328

Table S4: Comparison of orbitals and their respective energies involved in electronic transitions for S_1 state with their distribution in the S_0 state.

Orbital	S_0	S_1

HOMO	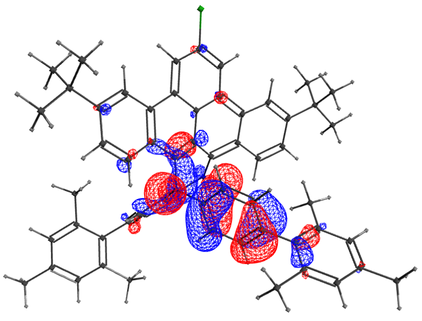 -5.831 eV	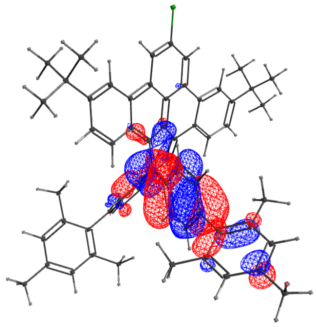 -5.288 eV
LUMO	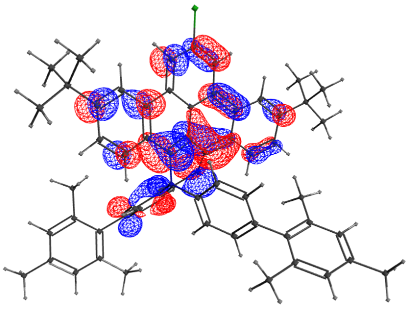 -2.589 eV	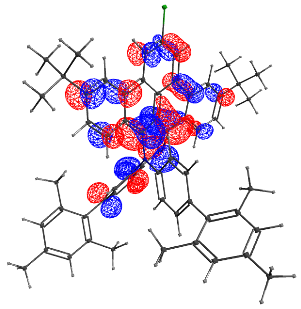 -2.932 eV
LUMO+1	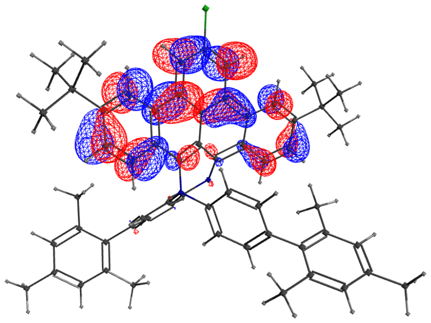 -1.801 eV	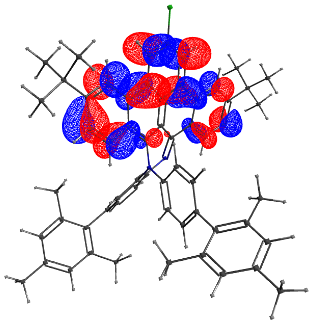 -1.853 eV
HOMO-1	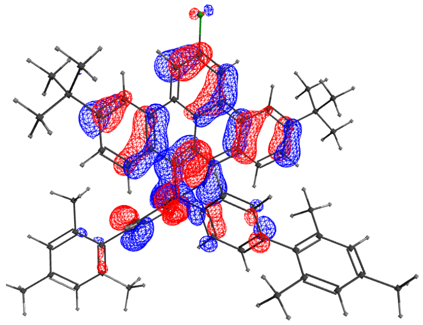 -6.028 eV	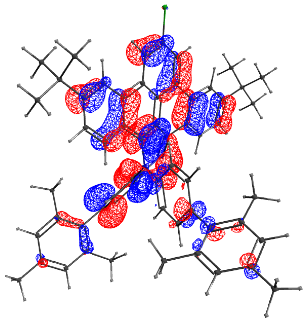 -5.881 eV
HOMO-2	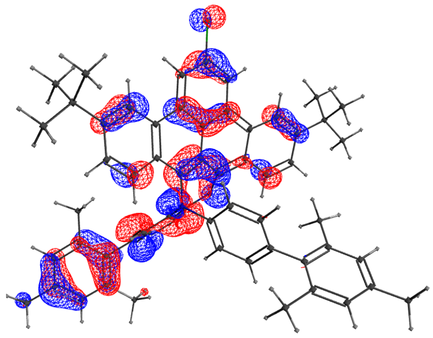	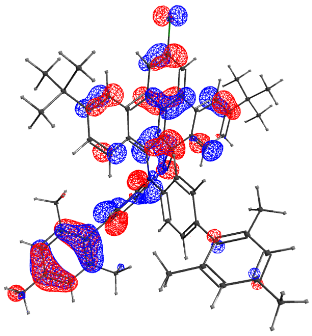

	-6.315 eV	-6.390 eV
HOMO-3	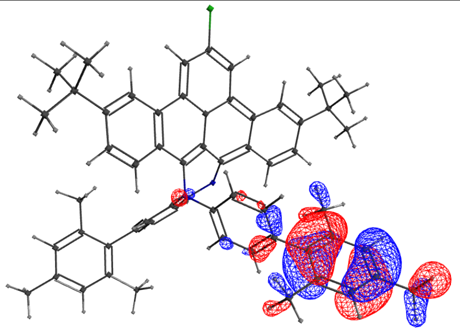 -6.458 eV	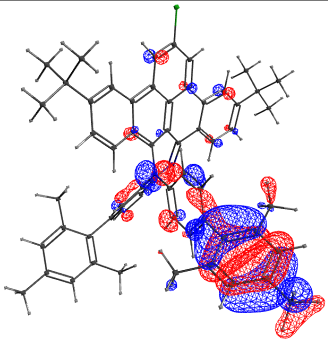 -6.503 eV
HOMO-5	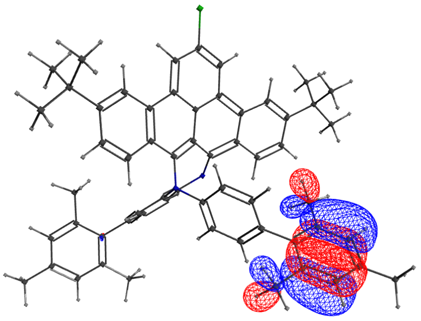 -6.581 eV	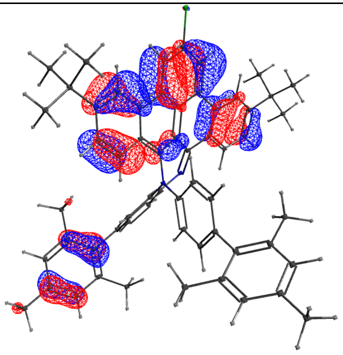 -6.603 eV
HOMO-7	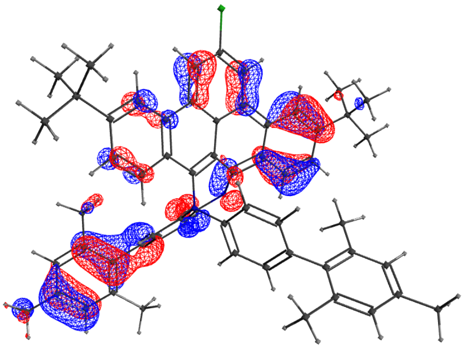 -6.685 eV	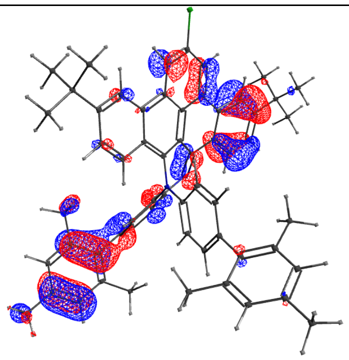 -6.686 eV
HOMO-8	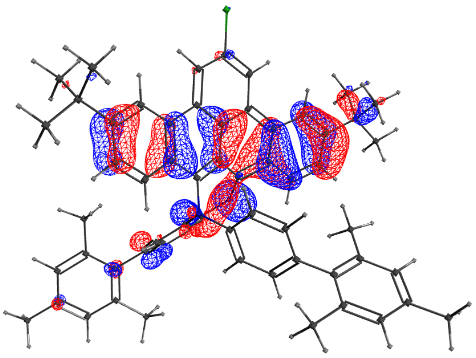	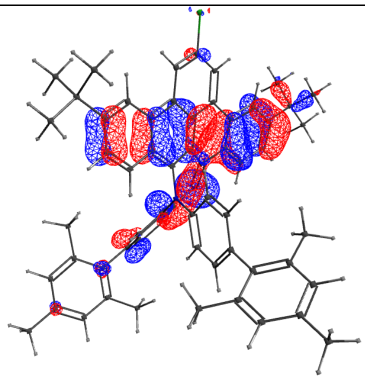

	-6.927 eV	-6.983 eV

Coordinates of optimized geometries (nroot=n-keyword). For running the calculations, using full Z-matrices is advised, since these tend to converge faster in comparison to cartesian coordinates.

^{Cl}BBTDZ (S_0)

C 2.08549 -1.60511 2.17682
C 0.96038 -0.90076 1.76773
C 2.34868 -2.89548 1.70673
C 0.06314 -1.47059 0.85398
C 0.28676 -2.78182 0.41969
N -1.04532 -0.72162 0.38674
C 1.41663 -3.47589 0.84349
C -0.88825 0.70174 0.33003
N 0.25688 -0.55542 -2.18508
C -1.70915 -1.16174 -0.80368
C -1.02999 -1.04562 -2.03546
C -1.68626 -1.51787 -3.18799
C -2.99205 -1.69327 -0.73960
C -3.65063 -2.11957 -1.89198
C -2.97388 -2.02418 -3.11823
H -3.47036 -2.35320 -4.02374
C 0.08250 1.23038 -0.48665
C -1.72958 1.53674 1.13119
C 0.77401 0.41356 -1.48991
C 0.41625 2.62693 -0.38667
C 2.56431 2.14954 -1.56213
C 3.89025 2.49743 -1.84774
C 4.81163 1.58491 -2.36496
C 4.36754 0.27765 -2.61022
C 3.05829 -0.09151 -2.34063
H 4.22241 3.50422 -1.63564
C 6.25662 2.03093 -2.61939
C -0.43012 3.50023 0.34719
C -0.13089 4.87604 0.35984
C 0.98569 5.33643 -0.30420
C 1.87009 4.48046 -0.95909
C 1.60889 3.11515 -0.99011
H -0.75754 5.57928 0.88694
H 2.74981 4.89250 -1.43134
C -1.55411 2.94796 1.09245
C -2.44718 3.74769 1.82658
C -3.47412 3.21623 2.59737
C -3.60224 1.81296 2.64717
C -2.74926 0.99456 1.93661
H -2.33147 4.82161 1.79609
C -4.41229 4.15797 3.36311
H -4.37809 1.35842 3.24698
C 7.12004 0.89995 -3.19949
C 6.88738 2.48725 -1.28597
C 6.26088 3.20671 -3.61966
H 7.18392 0.04869 -2.51696
H 8.13505 1.26837 -3.36656
H 6.73238 0.54546 -4.15797
H 5.81285 2.90722 -4.57077
H 7.28730 3.53179 -3.81162
H 5.70331 4.06475 -3.23842
H 6.88920 1.67010 -0.55976
H 6.34382 3.32730 -0.84848
H 7.92154 2.80364 -1.44906
C -5.48182 3.39427 4.15958
C -5.12747 5.08770 2.35916
C -3.58589 5.00771 4.35245
H -5.71410 4.50614 1.64316
H -4.41788 5.69907 1.79782
H -5.80523 5.76164 2.89060
H -6.11904 2.79112 3.50784
H -6.12254 4.10898 4.68152
H -5.03628 2.73759 4.91113
H -3.06471 4.36855 5.06997
H -4.24441 5.68099 4.90834
H -2.84009 5.61701 3.83773
C 2.14848 0.82462 -1.80842
H 5.04685 -0.46768 -2.99925
H -3.47254 -1.77460 0.22746
H 2.78313 -1.13605 2.86190
H 1.59007 -4.47922 0.47013
C 3.60860 -3.60259 2.07642
C 4.79368 -3.31326 1.37448

C 4.79915 -2.30579 0.24961
C 5.97099 -3.97972 1.72300
H 6.88194 -3.75965 1.17445
C 6.00471 -4.92202 2.75265
C 7.29478 -5.60308 3.13745
C 4.81759 -5.19681 3.43337
H 4.82197 -5.93007 4.23422
C 3.61985 -4.55263 3.11186
C 2.35761 -4.87826 3.87274
C -5.03718 -2.66066 -1.81561
C -5.26746 -4.04015 -1.96446
C -4.12442 -5.00069 -2.18941
C -6.57567 -4.52287 -1.88271
H -6.74882 -5.58957 -1.98676
C -7.66098 -3.67295 -1.66422
C -9.06958 -4.21046 -1.61807
C -7.40897 -2.30827 -1.51496
H -8.23943 -1.63081 -1.34145
C -6.11510 -1.78649 -1.58357
C -5.89438 -0.30174 -1.41585
H 4.04548 -2.54673 -0.50409
H 5.77491 -2.27140 -0.23789
H 4.56385 -1.30001 0.60791
H 1.89979 -3.97507 4.28487
H 2.56506 -5.56587 4.69434
H 1.60895 -5.34044 3.22296
H 7.95806 -5.71327 2.27656
H 7.11095 -6.59397 3.55851
H 7.83347 -5.02083 3.89289
H -3.68050 -4.87104 -3.18051
H -4.46661 -6.03355 -2.10800
H -3.32383 -4.84330 -1.46208
H -5.39377 -0.07357 -0.47042
H -6.84544 0.23294 -1.42900
H -5.26128 0.09943 -2.21118
H -9.09048 -5.23891 -1.25105
H -9.51952 -4.20983 -2.61672
H -9.70640 -3.60170 -0.97251
Cl 1.33378 7.06480 -0.30040
H -0.39993 -3.24595 -0.27596
H 0.79405 0.10402 2.13430
H -2.85501 -0.08099 1.97869
H -1.16640 -1.45151 -4.13611
H 2.72471 -1.10540 -2.51908

^{Cl}**BBTDZ** (S_1)
C 1.23840 2.92383 1.35630
C 0.67644 1.71538 1.02502
C 2.28752 3.48393 0.59657
C 1.14389 0.99879 -0.11180
C 2.17380 1.57733 -0.90563
N 0.58812 -0.19892 -0.44806
C 2.73062 2.77978 -0.54258
C -0.50037 -0.76057 0.29649
N -1.17196 -0.69080 -2.68846
C 1.02898 -0.96201 -1.56718
C 0.09658 -1.17179 -2.61431
C 0.60443 -1.89182 -3.72876
C 2.32795 -1.46107 -1.59716
C 2.78183 -2.20504 -2.68255
C 1.89019 -2.39836 -3.75391
H 2.21958 -2.96817 -4.61571
C -1.78459 -0.70664 -0.27410
C -0.23361 -1.29594 1.58184
C -2.01140 -0.45219 -1.66922
C -2.90502 -0.93040 0.61077
C -4.42665 -0.03281 -1.14004
C -5.67318 0.47620 -1.54735
C -5.89518 1.01823 -2.80756
C -4.80673 1.04188 -3.70074
C -3.56791 0.55524 -3.32881
H -6.49525 0.45695 -0.84545
C -7.28311 1.56273 -3.17072
C -2.69018 -1.44158 1.92785
C -3.79594 -1.67137 2.75798
C -5.07414 -1.41284 2.29088
C -5.30918 -0.90124 1.02859
C -4.22945 -0.63035 0.17608
H -3.67097 -2.04418 3.76293
H -6.32580 -0.71936 0.71447
C -1.32934 -1.70585 2.39623
C -1.05696 -2.31380 3.62988
C 0.23311 -2.50977 4.11734
C 1.29913 -2.06440 3.31380
C 1.07687 -1.47616 2.08483
H -1.88715 -2.65029 4.23494
C 0.44096 -3.18925 5.47635
H 2.32100 -2.18975 3.64499
C -7.33494 2.11222 -4.60508
C -7.65432 2.70578 -2.20169
C -8.32656 0.43139 -3.04964
H -6.63688 2.94137 -4.74648
H -8.34073 2.48440 -4.81572
H -7.10471 1.33935 -5.34292
H -8.08348 -0.39224 -3.72624
H -9.32147 0.80573 -3.30849
H -8.37150 0.03169 -2.03433
H -6.92838 3.52055 -2.26942
H -7.68029 2.36237 -1.16529
H -8.64232 3.10544 -2.44903
C 1.92820 -3.30586 5.84547
C -0.15946 -4.61113 5.43476

C -0.26615 -2.36842 6.57642
H 0.32680 -5.21300 4.66244
H -1.23030 -4.58994 5.22125
H -0.01812 -5.10973 6.39827
H 2.48006 -3.90633 5.11767
H 2.02405 -3.79301 6.81901
H 2.40589 -2.32510 5.91593
H 0.14231 -1.35540 6.62517
H -0.12431 -2.84267 7.55201
H -1.34016 -2.29102 6.39426
C -3.33761 0.02567 -2.04480
H -4.92538 1.45113 -4.69486
H 2.98716 -1.26646 -0.75964
H 0.88147 3.44986 2.23158
H 3.50007 3.21526 -1.16604
C 2.89072 4.77993 0.97191
C 2.09116 5.94278 1.02544
C 0.62153 5.92290 0.67543
C 2.68822 7.15529 1.36902
H 2.07816 8.05258 1.38201
C 4.04431 7.24842 1.68519
C 4.65522 8.56204 2.09799
C 4.81460 6.08520 1.63305
H 5.86918 6.13617 1.88396
C 4.26914 4.85421 1.27225
C 5.15970 3.63376 1.25461
C 4.15774 -2.77462 -2.69320
C 5.14360 -2.22907 -3.53685
C 4.83697 -1.05539 -4.43599
C 6.42982 -2.77368 -3.52338
H 7.18931 -2.34228 -4.16834
C 6.76479 -3.84915 -2.69913
C 8.14961 -4.44656 -2.72809
C 5.77440 -4.37333 -1.86724
H 6.01438 -5.21004 -1.21823
C 4.47743 -3.85448 -1.84834
C 3.44098 -4.46353 -0.93396
H 0.41305 5.27258 -0.17603
H 0.27846 6.92871 0.42953
H 0.01341 5.56630 1.51184
H 4.66210 2.76055 1.68086
H 6.06940 3.82152 1.82644
H 5.46208 3.36978 0.23705
H 4.11494 9.40517 1.66316
H 5.70202 8.62635 1.79398
H 4.62254 8.67657 3.18675
H 4.17145 -1.33960 -5.25598
H 5.75308 -0.65402 -4.87245
H 4.33457 -0.25480 -3.88707
H 3.19808 -3.79957 -0.09915
H 3.80105 -5.40557 -0.51716
H 2.50516 -4.65385 -1.46493
H 8.90042 -3.69684 -2.98742
H 8.21379 -5.24577 -3.47435
H 8.41590 -4.88016 -1.76164
Cl -6.45036 -1.73951 3.35147
H 2.49630 1.07949 -1.80699
H -0.11896 1.30081 1.62442
H 1.92654 -1.16756 1.49066
H -0.06732 -2.04861 -4.56418
H -2.73856 0.57959 -4.02260

^{Cl}**BBTDZ** (S_2)
C 1.12105 -1.91744 2.33062
C 0.42182 -0.88776 1.71334
C 0.92047 -3.25481 1.97573
C -0.50962 -1.16809 0.70135
C -0.73210 -2.50754 0.35186
N -1.20748 -0.11271 0.06078
C -0.02589 -3.52589 0.98477
C -0.55217 1.16355 0.06096
N 0.03873 -0.24332 -2.54259
C -1.95574 -0.43069 -1.10627
C -1.26178 -0.47680 -2.36002
C -2.04506 -0.84250 -3.50486
C -3.30015 -0.70113 -1.02184
C -4.06050 -1.05973 -2.15724
C -3.38350 -1.13518 -3.39730
H -3.94290 -1.38743 -4.28898
C 0.60531 1.33650 -0.71765
C -1.00556 2.15921 0.94779
C 0.93091 0.43381 -1.78423
C 1.49096 2.42430 -0.40831
C 3.26122 1.32187 -1.75082
C 4.60526 1.16515 -2.13275
C 5.04738 0.11585 -2.92816
C 4.08330 -0.81295 -3.38018
C 2.75617 -0.69061 -3.02932
H 5.33100 1.88373 -1.77842
C 6.53446 0.00378 -3.28458
C 1.05599 3.46403 0.46649
C 1.92363 4.53324 0.72893
C 3.18178 4.56279 0.14066
C 3.63996 3.54843 -0.67742
C 2.80907 2.44740 -0.95041
H 1.63885 5.33299 1.39576
H 4.63015 3.61808 -1.10188
C -0.25619 3.37090 1.09855
C -0.79009 4.38751 1.89972
C -1.99421 4.25746 2.59057
C -2.68497 3.03039 2.47926
C -2.20589 2.00970 1.69008
H -0.24518 5.31646 1.99345

C -2.50857 5.42181 3.44264
H -3.61196 2.87965 3.01491
C 6.83104 -1.21607 -4.17113
C 7.35935 -0.12970 -1.98585
C 6.97644 1.27336 -4.04437
H 6.56422 -2.15239 -3.67441
H 7.90033 -1.25077 -4.39336
H 6.29556 -1.16560 -5.12264
H 6.40203 1.39156 -4.96694
H 8.03612 1.20422 -4.30662
H 6.83745 2.17347 -3.44199
H 7.06118 -1.02161 -1.42852
H 7.22798 0.73615 -1.33360
H 8.42365 -0.21362 -2.22411
C -3.85027 5.10137 4.12002
C -2.70410 6.66336 2.54455
C -1.47468 5.74499 4.54422
H -3.43117 6.45830 1.75444
H -1.76924 6.97229 2.07243
H -3.07308 7.50253 3.14126
H -4.63220 4.88530 3.38748
H -4.17425 5.96383 4.70747
H -3.76853 4.24917 4.79948
H -1.31730 4.87899 5.19243
H -1.83135 6.57504 5.16089
H -0.50945 6.03112 4.12134
C 2.31252 0.35106 -2.18804
H 4.37922 -1.63956 -4.01102
H -3.76876 -0.66094 -0.04737
H 1.84354 -1.67445 3.10211
H -0.20396 -4.55319 0.68593
C 1.70884 -4.34812 2.61339
C 3.03517 -4.58144 2.20506
C 3.67182 -3.74183 1.12337
C 3.76205 -5.61259 2.80523
H 4.78213 -5.79456 2.48051
C 3.21011 -6.41601 3.80449
C 4.02475 -7.50014 4.46567
C 1.89238 -6.17024 4.19332
H 1.44342 -6.78561 4.96717
C 1.13256 -5.15058 3.61409
C -0.28865 -4.91988 4.06809
C -5.50309 -1.31789 -2.04473
C -6.06121 -2.52397 -2.54698
C -5.21312 -3.60968 -3.16688
C -7.42764 -2.75030 -2.40741
H -7.84000 -3.68945 -2.76241
C -8.27910 -1.81791 -1.80878
C -9.75977 -2.07076 -1.71433
C -7.71826 -0.63362 -1.32640
H -8.36534 0.11029 -0.87260
C -6.35473 -0.36612 -1.42200
C -5.84864 0.96367 -0.91287
H 3.04188 -3.69764 0.23124
H 4.64493 -4.14712 0.84095
H 3.81793 -2.70923 1.45267
H -0.45583 -3.87192 4.32998
H -0.52267 -5.53587 4.93803
H -1.00490 -5.16378 3.27821
H 4.76702 -7.91378 3.77931
H 3.38964 -8.31621 4.81736
H 4.56504 -7.10790 5.33409
H -5.02025 -3.41829 -4.22650
H -5.72610 -4.57044 -3.09982
H -4.24402 -3.70001 -2.67395
H -5.44466 0.88516 0.10095
H -6.66538 1.68655 -0.87932
H -5.05606 1.36739 -1.54426
H -9.97413 -3.13531 -1.59769
H -10.26720 -1.73391 -2.62520
H -10.20307 -1.53222 -0.87437
Cl 4.23675 5.93843 0.46953
H -1.44395 -2.75430 -0.42410
H 0.60640 0.13617 2.00886
H -2.74698 1.07689 1.60686
H -1.54289 -0.87722 -4.46405
H 2.02657 -1.40706 -3.38313`

^{Cl}**BBTDZ** (S_3)
C 2.07487 -1.23153 2.17947
C 0.90199 -0.62864 1.74238
C 2.38441 -2.55271 1.84306
C 0.00088 -1.33572 0.93392
C 0.27503 -2.67324 0.63191
N -1.15000 -0.68263 0.42765
C 1.45135 -3.26555 1.08393
C -1.01650 0.72619 0.18072
N 0.07634 -0.82860 -2.13809
C -1.84006 -1.27338 -0.67682
C -1.20269 -1.25206 -1.95347
C -1.94200 -1.75300 -3.05200
C -3.13090 -1.75312 -0.52820
C -3.85172 -2.25079 -1.61985
C -3.22339 -2.24381 -2.88172
H -3.76805 -2.61134 -3.74332
C 0.02662 1.15092 -0.68281
C -1.82930 1.65537 0.85946
C 0.66371 0.22846 -1.60735
C 0.52246 2.47296 -0.55504
C 2.63169 1.76696 -1.65753
C 3.99873 1.95597 -1.92739
C 4.80584 0.96263 -2.47928
C 4.19323 -0.25957 -2.80448

C 2.84777 -0.47677 -2.55323
H 4.44827 2.90588 -1.67193
C 6.30186 1.22510 -2.68525
C -0.26564 3.45466 0.13896
C 0.19734 4.76870 0.17558
C 1.41330 5.09742 -0.42924
C 2.21879 4.16033 -1.05285
C 1.80364 2.82412 -1.10456
H -0.36539 5.54843 0.66609
H 3.15360 4.47272 -1.49394
C -1.51913 3.04996 0.77701
C -2.38101 3.96264 1.39332
C -3.51448 3.57458 2.11548
C -3.76174 2.19456 2.24228
C -2.94277 1.26038 1.64040
H -2.16135 5.01872 1.31829
C -4.41659 4.64251 2.74012
H -4.61159 1.84489 2.81205
C 7.02250 0.01652 -3.30240
C 6.95311 1.53097 -1.31852
C 6.48907 2.43504 -3.62534
H 6.95222 -0.86652 -2.66213
H 8.08203 0.25165 -3.42925
H 6.61789 -0.23753 -4.28551
H 6.03273 2.24180 -4.59974
H 7.55419 2.63139 -3.77836
H 6.03740 3.34031 -3.21389
H 6.82713 0.68888 -0.63329
H 6.51364 2.41490 -0.85136
H 8.02408 1.71440 -1.44480
C -5.61102 4.02971 3.48770
C -4.96224 5.56316 1.62612
C -3.59720 5.48474 3.74323
H -5.54732 4.98927 0.90275
H -4.15679 6.06611 1.08676
H -5.60896 6.33219 2.05834
H -6.24427 3.43765 2.82196
H -6.22571 4.82983 3.90707
H -5.28753 3.39164 4.31404
H -3.19720 4.85400 4.54138
H -4.23311 6.25045 4.19689
H -2.75904 5.98975 3.25841
C 2.05007 0.50310 -1.94224
H 4.77336 -1.06334 -3.23606
H -3.56755 -1.75729 0.46233
H 2.77107 -0.65851 2.78174
H 1.66226 -4.29356 0.81060
C 3.68920 -3.15627 2.23619
C 4.86354 -2.76789 1.56218
C 4.82152 -1.75809 0.44045
C 6.08220 -3.34234 1.93222
H 6.98337 -3.04873 1.40222
C 6.17024 -4.28573 2.95710
C 7.50206 -4.86486 3.36531
C 4.99459 -4.65855 3.61087
H 5.04120 -5.39257 4.40966
C 3.75610 -4.11115 3.26679
C 2.51225 -4.54263 4.00562
C -5.23772 -2.74674 -1.45143
C -5.55569 -4.09189 -1.74823
C -4.50073 -5.07124 -2.20459
C -6.86618 -4.53580 -1.57639
H -7.09927 -5.57523 -1.78502
C -7.88120 -3.68605 -1.13103
C -9.29846 -4.17767 -0.99318
C -7.54937 -2.36051 -0.84213
H -8.32620 -1.68316 -0.50134
C -6.24963 -1.87556 -0.98581
C -5.97174 -0.42393 -0.67170
H 4.02386 -1.97826 -0.27131
H 5.76920 -1.74003 -0.10031
H 4.63101 -0.74715 0.81202
H 1.93751 -3.67964 4.35119
H 2.76880 -5.15720 4.87021
H 1.84684 -5.12613 3.36286
H 8.18970 -4.91514 2.51805
H 7.38922 -5.87041 3.77652
H 7.97684 -4.24736 4.13552
H -4.24770 -4.93060 -3.25944
H -4.85581 -6.09618 -2.08581
H -3.57499 -4.95633 -1.63711
H -5.51239 -0.30214 0.31310
H -6.90084 0.14847 -0.67260
H -5.28830 0.02217 -1.39643
H -9.32813 -5.24382 -0.75851
H -9.85088 -4.03287 -1.92817
H -9.83357 -3.63549 -0.21069
Cl 1.94179 6.77031 -0.37661
H -0.41077 -3.23826 0.01354
H 0.69252 0.39959 2.00777
H -3.15039 0.20422 1.74252
H -1.47720 -1.74117 -4.03026
H 2.39019 -1.42534 -2.80382

^{Cl}**BBTDZ** (S_4)
C 1.84933 -1.39739 2.26582
C 0.75006 -0.69300 1.79307
C 2.06527 -2.73371 1.91521
C -0.17206 -1.30730 0.93232
C -0.00221 -2.66466 0.63216
N -1.22961 -0.54900 0.36872
C 1.10266 -3.35808 1.11782
C -0.95198 0.84330 0.17381

N 0.07685 -0.74565 -2.20555
C -1.90617 -1.10621 -0.76587
C -1.21153 -1.15790 -2.00218
C -1.89721 -1.76057 -3.08511
C -3.19643 -1.59126 -0.63805
C -3.87727 -2.14899 -1.72755
C -3.19394 -2.22951 -2.95078
H -3.69626 -2.65751 -3.81091
C 0.11738 1.19533 -0.66257
C -1.68281 1.82062 0.89198
C 0.69553 0.25892 -1.59186
C 0.66115 2.51768 -0.55978
C 2.71920 1.73909 -1.68694
C 4.09274 1.89090 -1.98405
C 4.86225 0.86946 -2.52984
C 4.20851 -0.34121 -2.81826
C 2.85635 -0.51832 -2.54529
H 4.57230 2.83119 -1.75043
C 6.36204 1.08315 -2.76601
C -0.06185 3.53275 0.14232
C 0.46936 4.85480 0.18156
C 1.68097 5.12956 -0.42042
C 2.42610 4.13998 -1.05027
C 1.93772 2.81080 -1.10894
H -0.05805 5.64916 0.68623
H 3.36771 4.40210 -1.50881
C -1.28514 3.19418 0.82703
C -2.07560 4.15875 1.49491
C -3.21633 3.83424 2.24087
C -3.55088 2.47811 2.33139
C -2.80141 1.49507 1.67917
H -1.79033 5.19858 1.43012
C -4.02316 4.94619 2.91566
H -4.40647 2.16443 2.91304
C 7.02639 -0.14345 -3.41045
C 7.04936 1.35107 -1.40937
C 6.57304 2.29488 -3.69819
H 6.94653 -1.02855 -2.77418
H 8.08893 0.05904 -3.56477
H 6.58692 -0.37733 -4.38362
H 6.08832 2.12920 -4.66389
H 7.64105 2.45245 -3.87320
H 6.16634 3.21290 -3.26856
H 6.90709 0.50563 -0.73106
H 6.64804 2.24396 -0.92493
H 8.12347 1.49935 -1.55334
C -5.22666 4.39366 3.69433
C -4.54594 5.91904 1.83577
C -3.11568 5.71400 3.90189
H -5.19326 5.39739 1.12613
H -3.72917 6.37822 1.27442
H -5.12413 6.72118 2.30295
H -5.92133 3.86017 3.04042
H -5.77199 5.22136 4.15360
H -4.91554 3.71648 4.49397
H -2.72919 5.04386 4.67405
H -3.68452 6.50969 4.39093
H -2.26444 6.17510 3.39621
C 2.09155 0.49125 -1.95239
H 4.75786 -1.16790 -3.24682
H -3.67177 -1.54114 0.33431
H 2.56826 -0.89125 2.90089
H 1.23683 -4.39715 0.83727
C 3.31323 -3.43960 2.32290
C 4.51623 -3.15760 1.64620
C 4.55569 -2.15986 0.51283
C 5.68323 -3.82580 2.02532
H 6.60646 -3.61328 1.49452
C 5.69173 -4.76134 3.06135
C 6.97087 -5.44328 3.47941
C 4.48856 -5.02862 3.71666
H 4.47334 -5.75550 4.52320
C 3.29974 -4.38441 3.36368
C 2.02169 -4.70406 4.10069
C -5.27728 -2.62757 -1.58073
C -5.58021 -3.99899 -1.70289
C -4.50012 -5.02149 -1.96480
C -6.90158 -4.42165 -1.54764
H -7.12580 -5.48114 -1.62504
C -7.93772 -3.52343 -1.28340
C -9.36297 -3.99897 -1.15924
C -7.61878 -2.16983 -1.16404
H -8.40999 -1.45452 -0.96124
C -6.30958 -1.70588 -1.30433
C -6.03005 -0.22681 -1.17983
H 3.77190 -2.35623 -0.22235
H 5.52087 -2.18783 0.00403
H 4.39341 -1.13849 0.86846
H 1.52392 -3.79311 4.44266
H 2.22099 -5.33684 4.96727
H 1.30948 -5.22774 3.45650
H 7.65349 -5.55948 2.63457
H 6.77511 -6.43156 3.90128
H 7.49358 -4.85840 4.24404
H -4.16093 -4.99274 -3.00417
H -4.86914 -6.02872 -1.76407
H -3.62184 -4.84153 -1.34072
H -5.53360 0.01409 -0.23497
H -6.95969 0.34333 -1.22113
H -5.37134 0.12290 -1.97791
H -9.40898 -5.01978 -0.77349
H -9.86080 -3.99550 -2.13510

H -9.94157 -3.35297 -0.49531
Cl 2.30988 6.77129 -0.38209
H -0.70630 -3.16500 -0.01931
H 0.62493 0.34834 2.05937
H -3.08292 0.45415 1.76542
H -1.38184 -1.82811 -4.03597
H 2.37341 -1.46051 -2.76755

^{Cl}**BBTDZ** (S_5)

C 1.84915 -1.39721 2.26598
C 0.74987 -0.69288 1.79317
C 2.06523 -2.73348 1.91529
C -0.17211 -1.30721 0.93229
C -0.00211 -2.66452 0.63202
N -1.22967 -0.54894 0.36864
C 1.10278 -3.35788 1.11775
C -0.95203 0.84334 0.17362
N 0.07669 -0.74569 -2.20565
C -1.90627 -1.10619 -0.76588
C -1.21168 -1.15792 -2.00224
C -1.89742 -1.76060 -3.08512
C -3.19650 -1.59129 -0.63799
C -3.87738 -2.14906 -1.72745
C -3.19412 -2.22955 -2.95071
H -3.69649 -2.65756 -3.81082
C 0.11738 1.19531 -0.66275
C -1.68285 1.82072 0.89170
C 0.69546 0.25884 -1.59200
C 0.66126 2.51760 -0.55991
C 2.71926 1.73883 -1.68706
C 4.09281 1.89051 -1.98415
C 4.86222 0.86904 -2.52999
C 4.20835 -0.34153 -2.81855
C 2.85619 -0.51853 -2.54558
H 4.57247 2.83074 -1.75046
C 6.36205 1.08257 -2.76610
C -0.06169 3.53274 0.14217
C 0.46969 4.85469 0.18153
C 1.68138 5.12931 -0.42035
C 2.42642 4.13972 -1.05023
C 1.93787 2.81060 -1.10901
H -0.05764 5.64909 0.68621
H 3.36808 4.40172 -1.50873
C -1.28509 3.19427 0.82674
C -2.07556 4.15891 1.49449
C -3.21635 3.83452 2.24036
C -3.55097 2.47841 2.33095
C -2.80152 1.49529 1.67884
H -1.79021 5.19872 1.42965
C -4.02317 4.94654 2.91504
H -4.40661 2.16477 2.91256
C 7.02627 -0.14409 -3.41054
C 7.04934 1.35037 -1.40943
C 6.57322 2.29431 -3.69823
H 6.94627 -1.02920 -2.77429
H 8.08885 0.05826 -3.56478
H 6.58684 -0.37789 -4.38374
H 6.08852 2.12872 -4.66396
H 7.64126 2.45176 -3.87320
H 6.16661 3.21236 -3.26859
H 6.90694 0.50493 -0.73113
H 6.64812 2.24330 -0.92497
H 8.12348 1.49851 -1.55335
C -5.22669 4.39410 3.69373
C -4.54591 5.91933 1.83509
C -3.11568 5.71439 3.90124
H -5.19322 5.39765 1.12546
H -3.72913 6.37849 1.27373
H -5.12411 6.72151 2.30221
H -5.92134 3.86054 3.03986
H -5.77205 5.22185 4.15290
H -4.91561 3.71700 4.49345
H -2.72923 5.04429 4.67345
H -3.68451 6.51013 4.39022
H -2.26442 6.17543 3.39554
C 2.09149 0.49107 -1.95259
H 4.75761 -1.16824 -3.24720
H -3.67179 -1.54119 0.33440
H 2.56799 -0.89106 2.90115
H 1.23708 -4.39691 0.83713
C 3.31321 -3.43930 2.32309
C 4.51609 -3.15779 1.64603
C 4.55541 -2.16059 0.51217
C 5.68309 -3.82594 2.02526
H 6.60624 -3.61380 1.49416
C 5.69168 -4.76093 3.06177
C 6.97080 -5.44282 3.47995
C 4.48861 -5.02771 3.71748
H 4.47346 -5.75417 4.52440
C 3.29981 -4.38355 3.36438
C 2.02183 -4.70265 4.10176
C -5.27737 -2.62765 -1.58052
C -5.58036 -3.99902 -1.70295
C -4.50035 -5.02150 -1.96533
C -6.90171 -4.42169 -1.54756
H -7.12597 -5.48116 -1.62519
C -7.93778 -3.52351 -1.28289
C -9.36302 -3.99905 -1.15857
C -7.61878 -2.16996 -1.16327
H -8.40992 -1.45467 -0.96014
C -6.30959 -1.70600 -1.30372
C -6.03002 -0.22696 -1.17890

H 3.77177 -2.35753 -0.22301
H 5.52066 -2.18851 0.00350
H 4.39277 -1.13911 0.86732
H 1.52455 -3.79149 4.44390
H 2.22109 -5.33554 4.96827
H 1.30921 -5.22598 3.45773
H 7.65381 -5.55832 2.63534
H 6.77510 -6.43143 3.90110
H 7.49303 -4.85833 4.24521
H -4.16159 -4.99272 -3.00483
H -4.86929 -6.02874 -1.76448
H -3.62183 -4.84156 -1.34159
H -5.53374 0.01375 -0.23390
H -6.95963 0.34322 -1.22026
H -5.37115 0.12287 -1.97679
H -9.40897 -5.01993 -0.77299
H -9.86102 -3.99541 -2.13434
H -9.94148 -3.35317 -0.49442
Cl 2.31051 6.77098 -0.38177
H -0.70607 -3.16487 -0.01957
H 0.62462 0.34844 2.05950
H -3.08311 0.45440 1.76516
H -1.38211 -1.82814 -4.03601
H 2.37314 -1.46064 -2.76793

References

(1) Killian, L.; Lutz, M.; Thevenon, A. A π-Extended β-Diketiminate Ligand via a Templated Scholl Approach. *Chem. Commun.* **2024**, *60* (52), 6663–6666. https://doi.org/10.1039/D4CC01627K.

(2) Fulmer, G. R.; Miller, A. J. M.; Sherden, N. H.; Gottlieb, H. E.; Nudelman, A.; Stoltz, B. M.; Bercaw, J. E.; Goldberg, K. I. NMR Chemical Shifts of Trace Impurities: Common Laboratory Solvents, Organics, and Gases in Deuterated Solvents Relevant to the Organometallic Chemist. *Organometallics* **2010**, *29* (9), 2176–2179. https://doi.org/10.1021/om100106e.

(3) Parr, R. G.; Weitao, Y. *Density-Functional Theory of Atoms and Molecules*; Oxford University Press, 1994.

(4) Runge, E.; Gross, E. K. U. Density-Functional Theory for Time-Dependent Systems. *Phys. Rev. Lett.* **1984**, *52* (12), 997–1000. https://doi.org/10.1103/PhysRevLett.52.997.

(5) Jacquemin, D.; Wathelet, V.; Perpète, E. A.; Adamo, C. Extensive TD-DFT Benchmark: Singlet-Excited States of Organic Molecules. *J. Chem. Theory Comput.* **2009**, *5* (9), 2420–2435. https://doi.org/10.1021/ct900298e.

(6) Sarkar, R.; Boggio-Pasqua, M.; Loos, P.-F.; Jacquemin, D. Benchmarking TD-DFT and Wave Function Methods for Oscillator Strengths and Excited-State Dipole Moments. *J. Chem. Theory Comput.* **2021**, *17* (2), 1117–1132. https://doi.org/10.1021/acs.jctc.0c01228.

(7) Frisch, M. J.; Trucks, G. W.; Schlegel, H. B.; Scuseria, G. E.; Robb, M. A.; Cheeseman, J. R.; Scalmani, G.; Barone, V.; Petersson, G. A.; Nakatsuji, H.; Li, X.; Caricato, M.; Marenich, A. V.; Bloino, J.; Janesko, B. G.; Gomperts, R.; Mennucci, B.; Hratchian, H. P.; Ortiz, J. V.; Izmaylov, A. F.; Sonnenberg, J. L.; Williams; Ding, F.; Lipparini, F.; Egidi, F.; Goings, J.; Peng, B.; Petrone, A.; Henderson, T.; Ranasinghe, D.; Zakrzewski, V. G.; Gao, J.; Rega, N.; Zheng, G.; Liang, W.; Hada, M.; Ehara, M.; Toyota, K.; Fukuda, R.; Hasegawa, J.; Ishida, M.; Nakajima, T.; Honda, Y.; Kitao, O.; Nakai, H.; Vreven, T.; Throssell, K.; Montgomery Jr., J. A.; Peralta, J. E.; Ogliaro, F.; Bearpark, M. J.; Heyd, J. J.; Brothers, E. N.; Kudin, K. N.; Staroverov, V. N.; Keith, T. A.; Kobayashi, R.; Normand, J.; Raghavachari, K.; Rendell, A. P.; Burant, J. C.; Iyengar, S. S.; Tomasi, J.; Cossi, M.; Millam, J. M.; Klene, M.; Adamo, C.; Cammi, R.; Ochterski, J. W.; Martin, R. L.; Morokuma, K.; Farkas, O.; Foresman, J. B.; Fox, D. J. Gaussian 16 Rev. C.01, 2016.

(8) Vosko, S. H.; Wilk, L.; Nusair, M. Accurate Spin-Dependent Electron Liquid Correlation Energies for Local Spin Density Calculations: A Critical Analysis. *Can. J. Phys.* **1980**, *58* (8), 1200–1211. https://doi.org/10.1139/p80-159.

(9) Lee, C.; Yang, W.; Parr, R. G. Development of the Colle-Salvetti Correlation-Energy Formula into a Functional of the Electron Density. *Phys. Rev. B* **1988**, *37* (2), 785–789. https://doi.org/10.1103/PhysRevB.37.785.

(10) Becke, A. D. Density-functional Thermochemistry. III. The Role of Exact Exchange. *The Journal of Chemical Physics* **1993**, *98* (7), 5648–5652. https://doi.org/10.1063/1.464913.

(11) Stephens, P. J.; Devlin, F. J.; Chabalowski, C. F.; Frisch, M. J. Ab Initio Calculation of Vibrational Absorption and Circular Dichroism Spectra Using Density Functional Force Fields. *J. Phys. Chem.* **1994**, *98* (45), 11623–11627. https://doi.org/10.1021/j100096a001.

(12) Grimme, S.; Ehrlich, S.; Goerigk, L. Effect of the Damping Function in Dispersion Corrected Density Functional Theory. *J. Comput. Chem.* **2011**, *32* (7), 1456–1465. https://doi.org/10.1002/jcc.21759.

(13) Ditchfield, R.; Hehre, W. J.; Pople, J. A. Self-Consistent Molecular-Orbital Methods. IX. An Extended Gaussian-Type Basis for Molecular-Orbital Studies of Organic Molecules. *J. Chem. Phys.* **1971**, *54* (2), 724–728. https://doi.org/10.1063/1.1674902.

(14) Hehre, W. J.; Ditchfield, R.; Pople, J. A. Self—Consistent Molecular Orbital Methods. XII. Further Extensions of Gaussian—Type Basis Sets for Use in Molecular Orbital Studies of Organic Molecules. *J. Chem. Phys.* **1972**, *56* (5), 2257–2261. https://doi.org/10.1063/1.1677527.

(15) Hariharan, P. C.; Pople, J. A. The Influence of Polarization Functions on Molecular Orbital Hydrogenation Energies. *Theoret. Chim. Acta* **1973**, *28* (3), 213–222. https://doi.org/10.1007/BF00533485.

(16) Francl, M. M.; Pietro, W. J.; Hehre, W. J.; Binkley, J. S.; Gordon, M. S.; DeFrees, D. J.; Pople, J. A. Self-consistent Molecular Orbital Methods. XXIII. A Polarization-type Basis Set for Second-row Elements. *J. Chem. Phys.* **1982**, *77* (7), 3654–3665. https://doi.org/10.1063/1.444267.

(17) Gordon, M. S.; Binkley, J. S.; Pople, J. A.; Pietro, W. J.; Hehre, W. J. Self-Consistent Molecular-Orbital Methods. 22. Small Split-Valence Basis Sets for Second-Row Elements. *J. Am. Chem. Soc.* **1982**, *104* (10), 2797–2803. https://doi.org/10.1021/ja00374a017.

(18) Clark, T.; Chandrasekhar, J.; Spitznagel, G. W.; Schleyer, P. V. R. Efficient Diffuse Function-Augmented Basis Sets for Anion Calculations. III. The 3-21+G Basis Set for First-Row Elements, Li–F. *J. Comput. Chem.* **1983**, *4* (3), 294–301. https://doi.org/10.1002/jcc.540040303.

(19) Spitznagel, G. W.; Clark, T.; von Ragué Schleyer, P.; Hehre, W. J. An Evaluation of the Performance of Diffuse Function-Augmented Basis Sets for Second Row Elements, Na-Cl. *J. Comput. Chem.* **1987**, *8* (8), 1109–1116. https://doi.org/10.1002/jcc.540080807.

(20) Tomasi, J.; Mennucci, B.; Cammi, R. Quantum Mechanical Continuum Solvation Models. *Chem. Rev.* **2005**, *105* (8), 2999–3094. https://doi.org/10.1021/cr9904009.

(21) Cossi, M.; Barone, V. Analytical Second Derivatives of the Free Energy in Solution by Polarizable Continuum Models. *J. Chem. Phys.* **1998**, *109* (15), 6246–6254. https://doi.org/10.1063/1.477265.

(22) Chemcraft - Graphical Software for Visualization of Quantum Chemistry Computations. https://www.chemcraftprog.com.

(23) Hanwell, M. D.; Curtis, D. E.; Lonie, D. C.; Vandermeersch, T.; Zurek, E.; Hutchison, G. R. Avogadro: An Advanced Semantic Chemical Editor, Visualization, and Analysis Platform. *J. Cheminform.* **2012**, *4* (1), 17. https://doi.org/10.1186/1758-2946-4-17.

(24) Lu, T.; Chen, F. Multiwfn: A Multifunctional Wavefunction Analyzer. *J. Comput. Chem.* **2012**, *33* (5), 580–592. https://doi.org/10.1002/jcc.22885.

(25) Schreurs, A. M. M.; Xian, X.; Kroon-Batenburg, L. M. J. EVAL15: A Diffraction Data Integration Method Based on Ab Initio Predicted Profiles. *J. Appl. Cryst.* **2010**, *43* (1), 70–82. https://doi.org/10.1107/S0021889809043234.

(26) Sevvana, M.; Ruf, M.; Usón, I.; Sheldrick, G. M.; Herbst-Irmer, R. Non-Merohedral Twinning: From Minerals to Proteins. *Acta Cryst. D* **2019**, *75* (12), 1040–1050. https://doi.org/10.1107/S2059798319010179.

(27) Sheldrick, G. M. SHELXT – Integrated Space-Group and Crystal-Structure Determination. *Acta Cryst. A* **2015**, *71* (1), 3–8. https://doi.org/10.1107/S2053273314026370.

(28) Sheldrick, G. M. Crystal Structure Refinement with SHELXL. *Acta Cryst. C* **2015**, *71* (1), 3–8. https://doi.org/10.1107/S2053229614024218.

(29) Spek, A. L. PLATON SQUEEZE: A Tool for the Calculation of the Disordered Solvent Contribution to the Calculated Structure Factors. *Acta Cryst. C* **2015**, *71* (1), 9–18. https://doi.org/10.1107/S2053229614024929.

(30) Spek, A. L. Structure Validation in Chemical Crystallography. *Acta Cryst. D* **2009**, *65* (2), 148–155. https://doi.org/10.1107/S090744490804362X.

(31) Krause, L.; Herbst-Irmer, R.; Sheldrick, G. M.; Stalke, D. Comparison of Silver and Molybdenum Microfocus X-Ray Sources for Single-Crystal Structure Determination. *J. Appl. Cryst.* **2015**, *48* (1), 3–10. https://doi.org/10.1107/S1600576714022985.
